# Structure and Recognition of a Novel HIV-1 gp120-gp41 Interface Antibody that Caused MPER Exposure through Viral Escape

**DOI:** 10.1371/journal.ppat.1006074

**Published:** 2017-01-11

**Authors:** Constantinos Kurt Wibmer, Jason Gorman, Gabriel Ozorowski, Jinal N. Bhiman, Daniel J. Sheward, Debra H. Elliott, Julie Rouelle, Ashley Smira, M. Gordon Joyce, Nonkululeko Ndabambi, Aliaksandr Druz, Mangai Asokan, Dennis R. Burton, Mark Connors, Salim S. Abdool Karim, John R. Mascola, James E. Robinson, Andrew B. Ward, Carolyn Williamson, Peter D. Kwong, Lynn Morris, Penny L. Moore

**Affiliations:** 1 Centre for HIV and STIs, National Institute for Communicable Diseases (NICD), of the National Health Laboratory Service (NHLS), Johannesburg, South Africa; 2 Faculty of Health Sciences, University of the Witwatersrand, Johannesburg, South Africa; 3 Vaccine Research Center, National Institute of Allergy and Infectious Diseases, National Institutes of Health, Bethesda, Maryland, United States of America; 4 Department of Integrative Structural and Computational Biology, CHAVI-ID, IAVI Neutralizing Antibody Center and Collaboration for AIDS Vaccine Discovery (CAVD), The Scripps Research Institute, La Jolla, California, United States of America; 5 Institute of Infectious Disease and Molecular Medicine (IDM) and Division of Medical Virology, University of Cape Town and NHLS, Cape Town, South Africa; 6 Department of Pediatrics, Tulane University Medical Center, New Orleans, Louisiana, United States of America; 7 Department of Immunology and Microbial Science, CHAVI-ID and IAVI Neutralizing Antibody Centre, The Scripps Research Institute, La Jolla, California, United States of America; 8 Ragon Institute of Massachusetts General Hospital, MIT and Harvard, Cambridge, Massachusetts, United States of America; 9 Laboratory of Immunoregulation, National Institute of Allergy and Infectious Diseases, National Institutes of Health, Bethesda, Maryland, United States of America; 10 Centre for the AIDS Programme of Research in South Africa (CAPRISA), University of KwaZulu-Natal, Durban, South Africa; 11 Department of Epidemiology, Columbia University, New York, New York, United States of America; Miller School of Medicine, UNITED STATES

## Abstract

A comprehensive understanding of the regions on HIV-1 envelope trimers targeted by broadly neutralizing antibodies may contribute to rational design of an HIV-1 vaccine. We previously identified a participant in the CAPRISA cohort, CAP248, who developed trimer-specific antibodies capable of neutralizing 60% of heterologous viruses at three years post-infection. Here, we report the isolation by B cell culture of monoclonal antibody CAP248-2B, which targets a novel membrane proximal epitope including elements of gp120 and gp41. Despite low maximum inhibition plateaus, often below 50% inhibitory concentrations, the breadth of CAP248-2B significantly correlated with donor plasma. Site-directed mutagenesis, X-ray crystallography, and negative-stain electron microscopy 3D reconstructions revealed how CAP248-2B recognizes a cleavage-dependent epitope that includes the gp120 C terminus. While this epitope is distinct, it overlapped in parts of gp41 with the epitopes of broadly neutralizing antibodies PGT151, VRC34, 35O22, 3BC315, and 10E8. CAP248-2B has a conformationally variable paratope with an unusually long 19 amino acid light chain third complementarity determining region. Two phenylalanines at the loop apex were predicted by docking and mutagenesis data to interact with the viral membrane. Neutralization by CAP248-2B is not dependent on any single glycan proximal to its epitope, and low neutralization plateaus could not be completely explained by N- or O-linked glycosylation pathway inhibitors, furin co-transfection, or pre-incubation with soluble CD4. Viral escape from CAP248-2B involved a cluster of rare mutations in the gp120-gp41 cleavage sites. Simultaneous introduction of these mutations into heterologous viruses abrogated neutralization by CAP248-2B, but enhanced neutralization sensitivity to 35O22, 4E10, and 10E8 by 10-100-fold. Altogether, this study expands the region of the HIV-1 gp120-gp41 quaternary interface that is a target for broadly neutralizing antibodies and identifies a set of mutations in the gp120 C terminus that exposes the membrane-proximal external region of gp41, with potential utility in HIV vaccine design.

## Author Summary

Our understanding of which regions of the HIV-1 envelope are targets for broadly neutralizing antibodies (likely required for an HIV-1 vaccine) has expanded greatly in recent years, offering insights for vaccine design. For instance, much of solvent-exposed gp41 is now known to be targeted by antibodies at the gp120-gp41 interface. In this study, we isolated the neutralizing monoclonal antibody CAP248-2B, and used structural biology to characterize its epitope which spanned both the gp120-gp41 and gp41-gp41 interfaces in a manner distinct from other antibodies. CAP248-2B extends the interface target to include the gp120 C terminus, effectively encircling the base of native pre-fusion trimers. While CAP248-2B recapitulated the donor’s plasma breadth, it had poor potency against some isolates as a consequence of low neutralization plateaus. Unlike many broadly neutralizing antibodies, these plateaus did not appear to be a consequence of glycan heterogeneity, suggesting additional mechanisms that contribute towards incomplete neutralization. Finally, we characterized viral escape pathways from CAP248-2B, and identified a cluster of unusual mutations in the gp160 cleavage sites that made HIV-1 viruses more sensitive to antibodies targeting highly conserved membrane-proximal epitopes. These mutations might improve the immunogenicity of gp41, and thereby inform HIV-1 immunogen design.

## Introduction

The HIV-1 envelope glycoprotein trimer (Env) is the only known target for neutralizing antibodies and is thus a focus for vaccine design efforts. However, the development of an effective HIV-1 vaccine has been thwarted by the complex nature of Env, and the inability to produce soluble Env immunogens able to elicit broadly neutralizing antibodies (bNAbs) [1]. Env is expressed as a single gp160 protomer that is extensively glycosylated and trimerized in the endoplasmic reticulum [2, 3]. These gp160 oligomers are cleaved into gp120 (receptor binding subunit) and gp41 (transmembrane subunit), resulting in a trimer of heterodimers that is subjected to extensive glycan processing in the Golgi apparatus [3, 4]. The cleaving of gp160 occurs primarily through furin activity at position R511, but a fraction of Env is also cleaved at position R504 [4–6]. During this process gp120 is often shed from non-covalently associated gp120-gp41 trimers, and the entry-competent form of Env may comprise only a small portion of the total Env content on the viral membrane [3, 4, 7]. The remainder often exists as gp41 “stumps”, and incorrectly processed or prematurely triggered monomers and oligomers. The abundance of these aberrant forms of Env on the viral surface, and the consequent exposure of immunodominant regions not normally present on entry competent trimers, misdirects the humoral immune response toward non-protective epitopes [8]. In addition Env is highly sequence variable, particularly within the V1-V5 variable loop regions, and heterogeneously glycosylated (even at conserved NxS/T sequons) because the densely packed glycans afford each other varied protection from glycan processing enzymes. This combination of factors favours the formation of strain-specific neutralizing antibodies [9–13].

Nevertheless most people develop some level of cross-neutralizing antibodies [14], and after several years of infection these can mature into HIV-1 bNAbs that are able to target more conserved regions of Env, and thus neutralize diverse viral strains [15–17]. In some instances these bNAbs can neutralize 50%—99% of globally circulating strains, and have been shown to prevent infection in animal models [18–24]. HIV-1 bNAbs often display unusual characteristics such as high levels of somatic hypermutation, unusually long complementarity determining regions (CDRs), and insertions/deletions in both the CDRs and antibody framework regions [25–27]. These features enable HIV-1 bNAbs to access epitopes that are often recessed or contain conserved glycan or membrane components. Interestingly, bNAbs that include glycan(s) in their epitope frequently display incomplete neutralization, usually because they have evolved to recognise a specific glycoform not present on every trimer [28–32]. More recently, incomplete neutralization has also been described for HIV-1 bNAbs with epitopes that do not appear to depend on glycan, suggesting that additional factors may contribute to neutralization potency [33, 34].

To understand how these unusual bNAb specificities might be elicited by an HIV-1 vaccine, there has been a concerted effort to isolate bNAbs from HIV-1 infected individuals and to define their targets and developmental pathways [29, 35–48]. The identification of bNAb-mediated immune selection pressures on Env, coupled with mutagenesis and structural biology techniques have been instrumental in our understanding of the epitopes susceptible to broad neutralization [49]. These include the CD4 binding site (CD4bs), a cluster of epitopes surrounding the N332 glycan, the membrane proximal external region of gp41 (MPER), and a number of quaternary structure specific epitopes in either the V2 trimer apex or the gp120-gp41 interface. The more recently discovered gp120-gp41 interface bNAbs 8ANC195, PGT151, VRC34, 35O22, and 3BC315 target distinct, usually glycan dependent epitopes, that overlap in gp41 [29, 44, 47, 48, 50]. For example, 8ANC195 requires glycans at positions N234 and N276, PGT151 at positions N611 or N637, and VRC34 and 35O22 at position N88. The identification of additional bNAbs that have epitopes within the gp120-gp41 interface could further enhance our understanding of this new site of vulnerability.

In a previous study we described CAP248, an HIV-1 subtype C infected participant in the CAPRISA 002 cohort who developed bNAbs to a trimer-specific epitope that could not be defined at the time [15]. Here, we have isolated a monoclonal antibody (mAb) that was representative of the broadly neutralizing plasma response, but lacked potency due to low neutralization plateaus that could not be accounted for by glycan heterogeneity. Through the interrogation of autologous selection pressure in Env, together with X-ray crystallography and electron microscopy, we mapped the CAP248-2B target to a glycan independent epitope in gp120 and gp41 that overlaps with previously identified gp120-gp41 interface bNAbs, but is distinct in its recognition of the gp160 cleavage motifs in the gp120 C terminus. Escape from this antibody conferred a viral phenotype that was exceptionally sensitive to neutralization by MPER directed antibodies, suggesting a role for the C terminus of gp120 in the exposure of important bNAb epitopes in gp41.

## Results

### Isolation of antibody CAP248-2B, which recapitulates CAP248 plasma neutralization breadth

**Fig 1:**
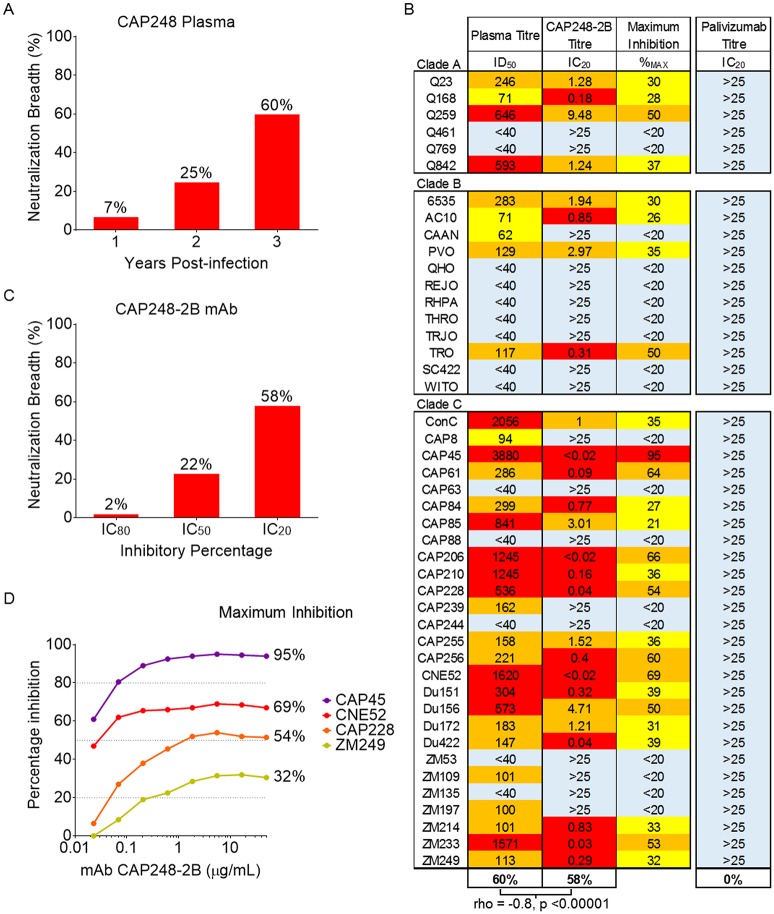
Isolated antibody CAP248-2B exhibits low neutralization plateaus, but recapitulates the plasma neutralization breadth. (A) Bar graph showing percentage neutralization breadth of CAP248 plasma at ID_50_ titers of >1:100 (y-axis) on a 45 virus panel at one, two, and three years post-infection (x-axis). The total breadth at each time point is indicated above the bars. (B) Comparison between the neutralization breadth of CAP248 plasma at three years, and CAP248-2B at IC_20_. The maximum percentage neutralization (neutralization plateau) reached by CAP248-2B is indicated. Palivizumab, a monoclonal antibody specific for Respiratory Syncytial Virus (RSV), was used as an IC_20_ negative control. Titers are colored yellow, orange and red by potency. A strong inverse spearman correlation with a rho value of -0.802 (p-value <0.00001) indicates good concordance between CAP248 plasma ID_50_ and CAP248-2B monoclonal antibody IC_20_. (C) Percentage neutralization breadth of monoclonal antibody CAP248-2B (y-axis) on the same 45 virus panel as in A, when measured at IC_80_, IC_50_, and IC_20_ (x-axis). (D) Neutralization curves of CAP248-2B against four viral strains (CAP45, CNE52, CAP228, and ZM249) plotted as percentage inhibition (y-axis) versus antibody concentration (x-axis). Dotted lines indicate y-axis intersections for IC_80_, IC_50_, and IC_20_. The maximum inhibitory percentage achieved against each virus is listed to the right of each curve.

CAP248 first developed cross-neutralizing antibodies after one year of infection. By three years, the neutralization breadth of CAP248 plasma increased incrementally, from 7% to 60% (Fig 1A) when tested against a 45 pseudovirus panel [15]. CAP248 plasma neutralized 78% of 27 subtype C, and 50% of 6 subtype A pseudoviruses, but only 25% of 12 subtype B pseudoviruses at titres >1:100 (Fig 1B). As mapping of polyclonal plasma may be complicated by the presence of multiple overlapping specificities [51, 52], we used a stored peripheral blood mononuclear cell (PBMC) sample from 3.5 years post-infection to isolate a monoclonal antibody, called CAP248-2B. B cells were enriched by negative selection with immunomagnetic beads, and B cell culture supernatants were screened after 14 days for neutralization of CAP45, a tier-2 pseudovirus sensitive to CAP248 plasma at ID_50_ titres of ~1:4,000. CAP248-2B was predicted to be derived from the IGHV4-31 and IGHJ3 heavy and IGLV2-14 and IGLJ1 lambda chain germline genes, and displayed modest levels of affinity maturation, with 13.5% and 9.7% nucleotide mutation in the heavy and light chains respectively (S1 Fig). CAP248-2B potently neutralized CAP45 with an IC_50_ of 0.04 μg/mL (IC_80_ of 0.19 μg/mL), but against the same panel on which CAP248 plasma displayed 60% neutralization breadth at three years post-infection, CAP248-2B neutralized only 22% of viruses with IC_50_ titres (Fig 1C).

Examination of the neutralization curves indicated that the poor breadth of CAP248-2B was due to incomplete neutralization, e.g. the maximum inhibition of CAP45, CNE52, CAP228, and ZM249 plateaued at 95%, 69%, 54%, and 32% respectively (Fig 1B and 1D). When IC_20_ values were examined, CAP248-2B neutralized a much larger fraction of the panel, showing 58% breadth, equivalent to the plasma breadth (Fig 1B and 1C). In contrast, weakly neutralizing antibodies 447-52D (that targets V3), 17b (co-receptor binding site), and HK20 (gp41), had sporadic, equivalent neutralization at both IC_20_ and IC_50_ (S2 Fig). No neutralization at IC_20_ was observed using Palivizumab (a negative control antibody) indicating that CAP248-2B IC_20_ titres were not the result of background activity in the assay (Fig 1B—right column). This phenomenon of neutralization at IC_20_ due to low neutralization plateaus has been observed for other HIV-1 antibodies, such as the PGT151-158 bNAb lineage [29]. For CAP248, there was significant concordance (p<0.00001) between pseudoviruses neutralized by the plasma at 3 years (measured as ID_50_) and those neutralized by the monoclonal antibody at IC_20_. This suggested that CAP248-2B was representative of the dominant bNAb specificity in CAP248 plasma, though more potent variants of this mAb lineage likely exist and remain to be isolated.

### Viral escape from CAP248-2B was mediated by unusual mutations in the gp160 cleavage site

Previously, we have shown that CAP248 plasma bNAbs could not be adsorbed using recombinant monomeric gp140 or MPER peptides, and were not sensitive to V2 mutations at positions N160 and L165 [15]. This suggested that CAP248 bNAbs targeted a quaternary epitope distinct from the V1V2 epitope. To identify potential escape mutations in response to CAP248 bNAbs, autologous gp160 sequences from nine weeks (study enrolment), as well as 1, 2, 3, and 3.5 years post-infection were examined for accumulating mutations (indicative of selection pressure) in normally conserved regions of the envelope.

**Fig 2:**
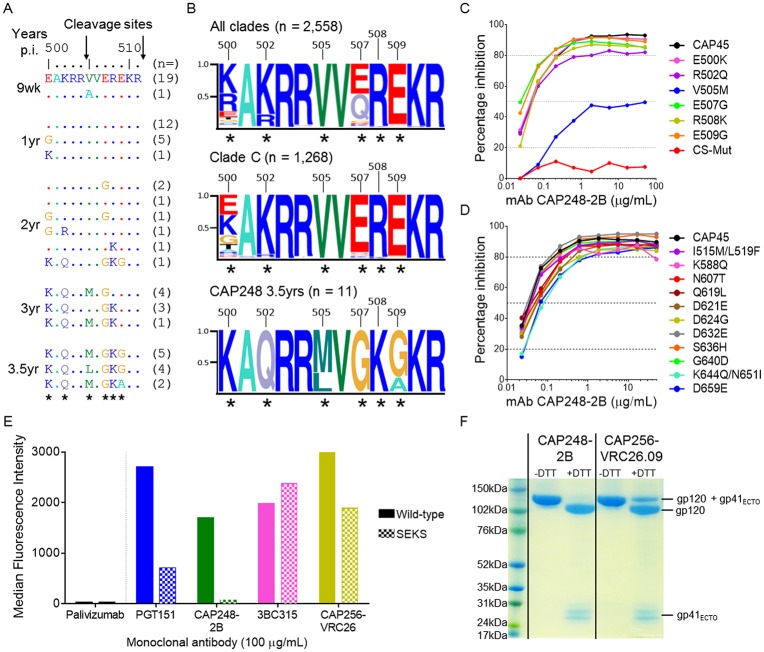
Escape mutations from CAP248-2B accumulate in the gp120 C terminus. (A) Sequence alignment of the gp120 C-terminus (positions 500–511) from CAP248 autologous viruses at nine weeks (study enrolment), 1, 2, 3 and 3.5 years post-infection. The primary (position 511) and secondary (position 504) gp160 cleavage sites are indicated with arrows. The total number of viruses with identical amino acid sequence within this region are indicated in brackets to the right. Residues undergoing significant selection pressure are indicated with the asterisks. (B) Sequence logograms showing variation within the gp120 C-terminus for all clades, and clade C only, from the LANL HIV sequence database, as well as from CAP248-2B at 3.5 years post-infection, colored and labelled as in A. The global frequencies for each of the autologous mutations identified in CAP248 sequences were: 500 (E8.88%, K46.56%, G3.56%), 502 (K79.35%, R18.73%, Q1.37%), 505 (V98.98%, A0.55%, M0.08%, L<0.01%), 507 (E47.5%, G3.87%, A0.7%), 508 (R98.24%, K1.49%), 509 (E95.11%, G1.53%, A0.27%). (C) Neutralization by CAP248-2B of the heterologous strain CAP45, when compared to gp120 C-terminal mutant viruses with changes identified from autologous CAP248 Env sequences. Data was plotted as percent inhibition (y-axis) against antibody concentration (x-axis). The wild-type virus is shown in black. Dotted lines indicate y-axis intersections for IC_80_, IC_50_, and IC_20_. (D) Neutralization by CAP248-2B of CAP45 wild-type and mutant viruses with the additional gp41 changes identified from CAP248 autologous sequences, plotted as in C. (E) Binding to cleaved (solid bars) or uncleaved (speckled bars) cell-surface expressed Env measured by flow cytometry. Median fluorescence intensity (MFI) is shown on the y-axis, and Palivizumab was used as an HIV-1 negative control. (F) An SDS-PAGE gel of a single SOSIP trimer sample that was divided into two and subsequently captured from suspension by either CAP248-2B or CAP256-VRC26.09. Samples were run in the presence or absence of dithiothreitol (DTT) to assess the level of furin cleavage.

Several autologous mutations were identified in, or proximal to, known bNAb epitopes within V2 (E164I/V, L165I/F), C1 (G87R/E) / C2 (D230N, N234T/S, T236K), V3 (D321E/G, N325D/K, E328K/D), and the MPER (N674G, K677N/Q, K683R) (S3A Fig). However when these changes were made in the heterologous virus CAP45, none affected CAP248-2B neutralization (S3B Fig). We also assessed the effect of mutations known to confer resistance to bNAbs targeting these four regions, but these mutations also failed to abrogate CAP248-2B neutralization (S3C Fig). There were slight effects following the T303A and F672L/W673L mutations, but these are also known to affect overall Env conformation [30, 53]. A cluster of unusual mutations were identified in the C terminus of gp120 at positions 500, 502, 505, 507, 508, and 509 within the gp160 furin cleavage motifs that separate gp120 from gp41 (Fig 2A). This region has not previously been implicated in viral escape from neutralizing antibodies, but analysis of 2,558 sequences from the Los Alamos National Laboratory HIV-1 database showed that while sequence variation at position 500 is common, mutations at the other five sites are rare, particularly amongst clade C viruses (Fig 2B). The simultaneous presence of these mutations in CAP248 viral sequences therefore suggested strong immune pressure on this region. When the six most common mutations at 3.5 years post-infection (E500K, R502Q, V505M, E507G, R508K, and E509G) were introduced individually into the heterologous virus CAP45, only the V505M mutation substantially reduced CAP248-2B neutralization (Fig 2C—blue line). However, the simultaneous introduction of all six mutations into CAP45, hereafter referred to as CAP45(CS-Mut), conferred complete resistance to both CAP248-2B neutralization (Fig 2C—red line), as well as CAP248 broadly neutralizing plasma (S3D Fig), confirming their collective role in escape from CAP248 bNAbs.

The gp120 C terminus is proximal to gp41, suggesting that the CAP248-2B epitope might be in the gp120-gp41 interface. To determine whether autologous gp41 mutations might contribute towards escape from CAP248-2B, we examined CAP248 gp41 ectodomain sequences over time. Of the thirteen changes identified (I515M, L519F, K588Q, N607T, Q619L, D621E, D624G, D632E, S636H, G640D, K644Q, N651I, and D659E), none affected CAP248-2B neutralization appreciably when introduced into CAP45, however these mutations may play a role in resistance to other members of the CAP248-2B lineage present in CAP248 plasma (Fig 2D). Altogether, these data suggest that major escape mutations from the CAP248-2B lineage accumulated in the gp160 cleavage motifs, with the role of additional mutations accumulating in proximal regions of gp41 still to be defined.

### CAP248-2B binds to a cleavage-dependent epitope

Antibodies such as PGT151 and VRC34 that recognize the gp41 N terminus bind only to fully cleaved Env [29, 50], while other gp120-gp41 interface bNAbs that do not recognize the peptide termini (35O22 and 3BC315) bind equally well to both cleaved and uncleaved Env [44, 48]. To assess the cleavage dependence of CAP248-2B, we compared the binding of CAP248-2B to cell surface expressed CAP45 envelope trimers, and an R508S/R511S mutant (SEKS mutant) [54] that is incompletely cleaved (Fig 2E). Both CAP248-2B and PGT151 bound less efficiently to the SEKS mutant Env, while 3BC315 and CAP256-VRC26.09 (a V2-directed bNAb) bound to both. CAP248-2B is escaped by mutations in the gp120 C terminus, and the R508S/R511S changes could directly cause resistance to CAP248-2B by altering contact residues in the gp120 C terminus. Therefore, to further confirm the role of cleavage in forming the CAP248-2B epitope, protein A coupled antibodies were used to capture soluble SOSIP trimers (described below) that were not co-transfected with furin (which is usually used to enhance cleavage efficacy). A single preparation of SOSIP trimer (containing both cleaved and uncleaved trimer) was divided into two, and passed over either CAP248-2B or CAP256-VRC29.09 antibody columns. SDS-PAGE showed that CAP248-2B was only able to capture completely cleaved SOSIP trimers, while CAP256-VRC26.09 could capture both cleaved and uncleaved Env from the same preparation (Fig 2F). These data confirm proper cleavage of Env is required for the formation of the CAP248-2B epitope.

### Structural characterization of monoclonal antibody CAP248-2B

**Fig 3:**
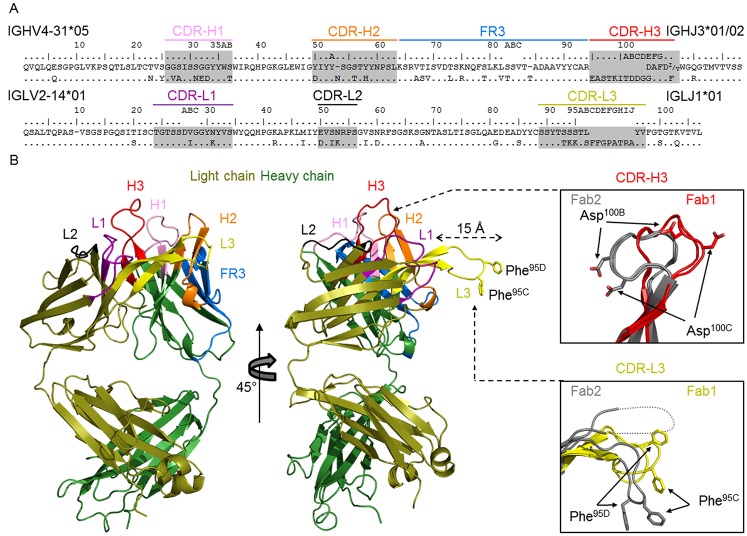
Crystal structure of CAP248-2B reveals an unusually long, protruding CDR-L3, with a hydrophobic tip. (A) Sequence alignment of the CAP248-2B heavy and light chain with their predicted V- and J-gene precursors. The CDRs are shaded, labelled, and colored. The heavy chain FR3 is similarly indicated in blue. (B) Crystal structure of the CAP248-2B Fab. The light and heavy chains are colored olive and forest green respectively, while CDR loops and FR-H3 are colored according to A. Two views are shown around a ~45° axis to highlight the long CDR-L3 (yellow). Insets show the conformational differences between the CDR-H3 and CDR-L3 loops between Fab structure 1 (shown here) and Fab structure 2 (shown in S1 Fig). Two Fabs were present in both asymmetric units, so four loops are shown per inset, two for Fab1 CDR-H3 (red) or L3 (yellow), and two for Fab2 CDR-H3 and L3 (both grey). Asp^100B^ and Asp^100C^ in the heavy chain and Phe^95C^ and Phe^95D^ in the light chain are shown with stick representations to highlight the conformational divergence between the two structures. Due to crystal packing all downstream analyses were based on the 3.1 Å Fab1 structure.

CAP248-2B possessed an average length CDR-H3 of 15 amino acids, but had an unusually long CDR-L3 of 19 amino acids (Fig 3A). The typical length of a CDR-L3 is 8–12 amino acids, and there are no antibodies with CDR-L3s of greater than 15 amino acids in the Abysis database (http://www.bioinf.org.uk/abysis/index.html). In addition to the CDRs, there was also substantial maturation away from germline in the framework region three (FR3) of the heavy chain (Fig 3A—blue). We determined two crystal structures of the unliganded CAP248-2B antigen binding fragment (Fab) to resolutions of 2.0 Å and 3.1 Å respectively (S1 Table and Fig 3B). The CDR-H3 and CDR-L3 loops of the 2.0 Å resolution structure were influenced slightly by crystal packing (S4A Fig), and a comparison of the CDR-H3 between the two structures revealed a potentially dynamic loop that flipped between two distinct conformations bent at residues Gly^100D^ and Gly^100E^ to flip the Asp^100B^ and Asp^100C^ anionic pair ~180° between CDR-L3 proximal and distal orientations (Fig 3B—top inset).

In the 3.1 Å resolution structure (not influenced by crystal packing), the 19 amino acid CDR-L3 formed a β-hairpin which was stabilized along its length by seven hydrogen bonds, and protruded ~15 Å at a right angle relative to the other CDR’s. The tip of the CDR-L3 ended in hydrophobic residues Phe^95C^ and Phe^95D^ that were angled by Pro^95F^ and immediately flanked by residues Ser^95B^ and Gly^95E^ which may confer a degree of plasticity to this region (Fig 3B—bottom inset). In both structures, the angle at which the CDR-L3 extended from the Fab was stabilized at its base by germline conserved hydrogen bonding interactions with CDR-L1, as well as a salt bridge formed between CDR-L3 residue Arg^95I^, and Asp^50^ in the CDR-H2. Overall, the CAP248-2B antigen binding site displayed substantial structural plasticity, an attribute that likely contributes to its mode of neutralization.

### CAP248-2B targets a membrane proximal epitope

**Fig 4:**
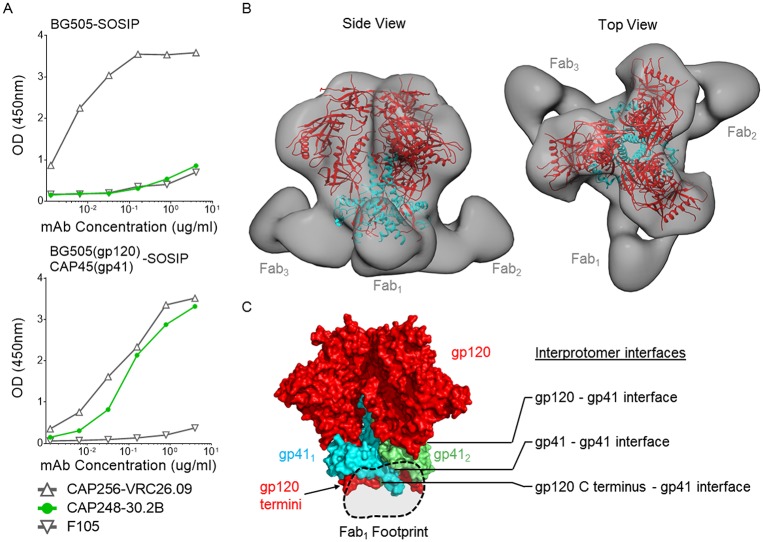
Negative-stain EM reveals a distinct membrane proximal epitope for CAP248-2B in the gp120 C terminus and parts of gp41. (A) ELISA data comparing the binding of trimer-specific antibodies CAP256-VRC26.09, CAP248-2B, and the non-neutralizing, gp120 binding antibody F105 to purified wild-type BG505 (top panel), and chimeric BG505(gp120)-CAP45(gp41) (bottom panel) SOSIP trimers. Absorbance readings are plotted on the y-axis and antibody concentration on the x-axis. (B) Negative stain EM 3D reconstruction of the CAP248-2B Fab bound trimers, shown in grey. The previously determined trimer structure with pdb ID: 4TVP was docked into the 3D reconstruction, and colored red for gp120 and cyan for gp41. Two views are shown: Perpendicular to the viral membrane (side view), and a view as seen from the target cell (top view). (C) A surface representation of the SOSIP trimer, where all gp120s are coloured red and two adjacent gp41s are shown in cyan and lime green. The gp120-gp41 interface, gp41-gp41 interface, and gp120 C terminus-gp41 interface are indicated, and the approximate CAP248-2B Fab footprint is shown with the dotted line.

To identify the binding site of CAP248-2B, we obtained structural information about the Fab bound to a soluble pre-fusion Env trimer by electron microscopy (EM) (Fig 4 and S4 Fig). CAP248-2B was unable to bind monomeric gp120, or gp145 proteins which contain the entire Env ectodomain (S4B Fig). Furthermore CAP248 plasma was unable to neutralize BG505, and consistent with these data CAP248-2B failed to bind recombinant BG505 SOSIP trimer in ELISA (Fig 4A—top graph). In the pre-fusion SOSIP structure, the gp120 C terminus is immediately proximal to gp41, and accordingly the CAP248-2B epitope could be engineered into the BG505 SOSIP trimer by designing a BG505(gp120)-CAP45(gp41) chimera. BG505 and CAP45 differed in gp41 by only 6.7% (23 amino acids), with most of these mutations not predicted to interact with gp120 in the pre-fusion structure. This chimeric SOSIP trimer was efficiently cleaved, and bound well to trimer-specific bNAbs CAP256-VRC26.09 and CAP248-2B, but not to the non-neutralizing antibody F105, suggesting that it retained a native-like pre-fusion conformation (Fig 4A—bottom graph). Single particle negative stain EM reconstructions at ~20 Å showed a maximum stoichiometry of three CAP248-2B Fabs bound to the BG505(gp120)-CAP45(gp41) SOSIP trimer (Fig 4B and S4C–S4F Fig). Docking of a SOSIP trimer crystal structure into the EM 3D reconstruction revealed a binding site for the CAP248-2B Fab that was extremely close to the viral membrane, similar to 35O22 and 3BC315 [44, 48], but approaching from an angle that was proximal to the gp120 C terminus (Fig 4B and 4C). The approximate Fab footprint bridged the gp41-gp41 interface, but did not encompass the gp120-gp41 interface bound by previously described bNAbs. Rather CAP248-2B binds to a second more membrane proximal interface between gp41 and the gp120 N- and C- termini (Fig 4C). Since this is also the site of viral escape mutations, these data support the hypothesis that CAP248-2B binds to the C terminus of gp120, as well as to parts of gp41.

### The CAP248-2B CDR-L3 interacts with the viral membrane

**Fig 5:**
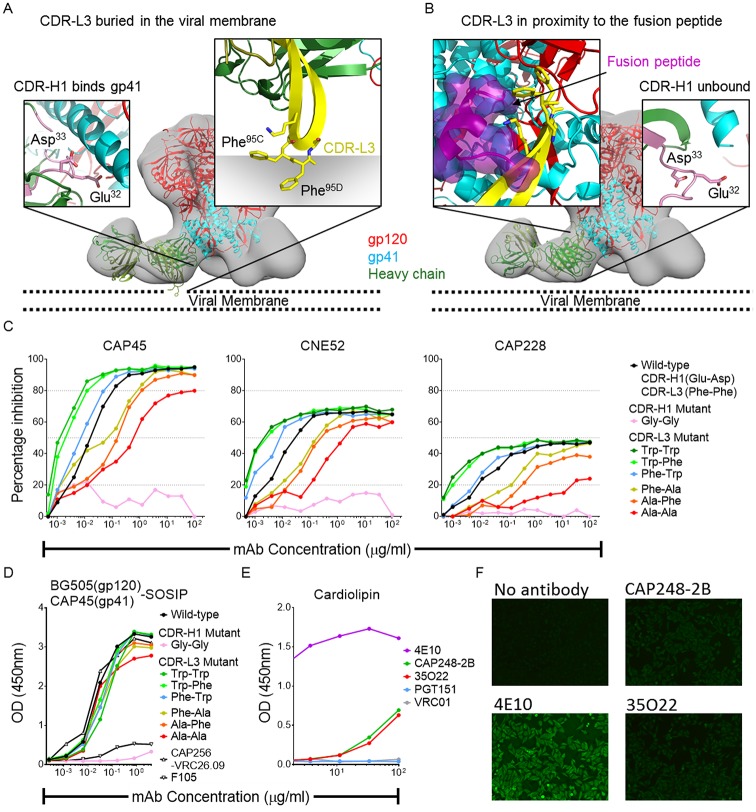
The CAP248-2B CDR-L3 interacts with the viral membrane. Two docking orientations for the CAP248-2B Fab are modelled with (A) the CDR-L3 in close proximity to the viral membrane, and (B) the CDR-L3 in close proximity to the fusion peptide. The trimer is coloured as in Fig 4, and the Fab heavy and light chains shown in forest and olive green respectively, and the approximate location of the viral membrane is indicated with dotted lines. In the zoomed panel insets, the CDR-H1 (pink) and CDR-L3 (yellow) are shown in their predicted binding locations for each model. The fusion peptide is colored purple and shown with surface representation. (C) Neutralization of three heterologous viruses by CAP248-2B and related CDR-H1 and CDR-L3 mutants. Percentage inhibition was plotted on the y-axis versus antibody concentration on the x-axis. Dotted lines indicate y-axis intersections for IC_80_, IC_50_, and IC_20_. (D) ELISA showing binding of CAP248-2B and related mutant antibodies to the BG505(gp120)-CAP45(gp41) chimeric SOSIP trimer. Absorbance readings are plotted on the y-axis and antibody concentration on the x-axis. CAP256-VRC26.09 and F105 are used are positive and negative control antibodies. (E) Anti-cardiolipin antibody ELISA, labelled as in D. (F) HEp-2 cell reactivity assays comparing a no antibody control to 50 μg/mL concentrations of either 4E10 (positive control), 35O22 (negative control), or CAP248-2B.

The CAP248-2B Fab structure could be docked into the EM reconstructions in two possible orientations. These placed the hydrophobic CDR-L3 tip in close proximity to either the viral membrane (Fig 5A—right inset), or the fusion peptide (FP) of gp41 (Fig 5B—left inset), with approximately 1,100 Å^2^–900 Å^2^ of surface area buried by the paratope in the Env SOSIP trimer respectively. The viral membrane bound model suggested a role for the heavy chain CDR-H1 in specific interactions with gp41, where two residues selected through somatic hypermutation, Glu^32^ and Asp^33^, are situated in close proximity to position N656 (Fig 5A—left inset). In the FP bound model, these residues do not interact with the SOSIP trimer which is truncated at Asp^664^ of gp41 (Fig 5B—right inset). When the CDR-H1 Glu^32^/Asp^33^ residue pair were reverted to germline (Gly^32^/Gly^33^), the mutant antibody failed to bind to SOSIP trimers or neutralize the CAP45 virus (Fig 5C and 5D –pink curves), providing evidence for the model where the CDR-L3 interacts with the viral membrane.

In this lipid binding model, Phe^95C^ and Phe^95D^ would insert into the core of the viral membrane, while Lys^94^ and Lys^95^ would be positioned to interact with the polar membrane lipid heads (Fig 5A). To provide further evidence for this binding orientation, we replaced the phenylalanine residues at the CAP248-2B CDR-L3 tip with either tryptophan or alanine (Fig 5C and 5D). Bulky, hydrophobic tryptophan side chains could interfere with specific protein-protein FP interactions, potentially impacting negatively on binding and neutralization. These same mutations would be expected to enhance interactions with the viral lipids by increasing the hydrophobicity of the CDR-L3, improving neutralization but not binding to soluble SOSIP trimers where the viral membrane is absent. The Trp^95C^/Trp^95D^ CDR-L3 mutant was substantially more potent against viruses CAP45, CNE52, and CAP228 in neutralization assays (Fig 5C). Similarly, mutating only one of the CDR-L3 Phe residues to generate Trp^95C^/Phe^95D^, or Phe^95C^/Trp^95D^ mutants, also resulted in enhanced neutralization, with Trp^95C^ showing the greater effect. In contrast the CDR-L3 Ala^95C^/Phe^95D^, Phe^95C^/Ala^95D^, and Ala^95C^/Ala^95D^ mutants all showed decreased neutralization potency against the same three viruses, confirming the importance of a hydrophobic CDR-L3 tip in effective neutralization of HIV-1. Despite these effects on neutralization, binding of CAP248-2B to soluble SOSIP trimers (in the absence of viral membrane) was not affected by CDR-L3 hydrophobicity (Fig 5D), supporting the docking model where the CDR-L3 enhances CAP248-2B neutralization by burying in the viral membrane. Other neutralizing antibodies that interact with the viral membrane (such as MPER antibodies 4E10 and 2F5) are often autoreactive [55], however CAP248-2B bound only very weakly to cardiolipin (Fig 5E) and HEp-2 epithelial cells (Fig 5F) compared to the 4E10 positive control, indicating no significant autoreactivity. Altogether, these data support a docking orientation where the CAP248-2B heavy chain makes contact with gp41, while the CDR-L3 makes productive hydrophobic interactions primarily with the viral membrane.

### The CAP248-2B binding site overlaps with several broadly neutralizing antibody epitopes in gp41

**Fig 6:**
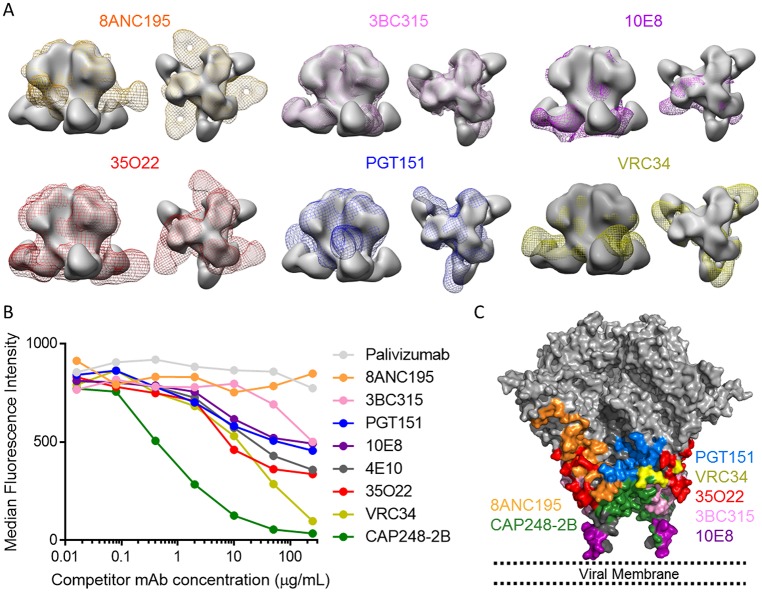
Broadly neutralizing antibodies that target gp41 compete with CAP248-2B for binding to cell-surface Env. (A) Comparisons of gp41 directed bNAbs bound to SOSIP trimers by EM. The CAP248-2B bound trimers are shown in solid grey surface, while 8ANC195, 3BC315, 10E8, 35O22, PGT151, and VRC34 bound trimers are shown with mesh representation. Both top views and side views are shown. (B) Binding of labelled CAP248-2B to cell-surface anchored HIV-1 Env by flow cytometry, in the presence of increasing concentrations of unlabeled competitor antibody. Median fluorescence intensity (MFI) is shown on the y-axis, and increasing concentrations of each competitor antibody is plotted on the x-axis. Decreasing MFI signals correspond to increasing competition with CAP248-2B. (C) Surface view of the envelope trimer with modelled MPER, colored to show the core epitopes for gp41 targeted bNAbs. The approximate location of the viral membrane is indicated.

A number of bNAbs target distinct epitopes in gp41, including the gp120-gp41 or gp41-gp41 interfaces, and the MPER. When the CAP248-2B EM reconstructions were superimposed with similar EM reconstructions of these antibodies, CAP248-2B bound in close proximity to the epitopes of almost all gp41 directed bNAbs (Fig 6A). These data suggested overlap with 35O22 and 3BC315 near the gp41-gp41 interface, with PGT151 and VRC34 near the fusion peptide, and with 10E8 near the MPER. We therefore assessed the ability of gp41 targeted antibodies to compete with CAP248-2B binding to cell surface expressed CAP45 envelope trimers by flow cytometry (Fig 6B). The gp120-gp41 interface antibodies VRC34, PGT151, and 35O22 (but not 8ANC195) competed for CAP248-2B binding, but 3BC315 only showed slight competition at the highest concentrations tested. In the reverse competition assay, CAP248-2B competed more substantially with 3BC315, consistent with the overlap observed by EM (S5 Fig). MPER bNAbs 4E10 and 10E8 also competed for CAP248-2B binding to Env, while CAP248-2B binding did not affect 4E10 in the reverse assay (Fig 6B and S5 Fig). Plotting the epitopes for these gp41 targeted antibodies, together with the CAP248-2B footprint, onto a model of the HIV-1 Env trimer with MPER (shown in the 10E8 bound form) shows how the distinct CAP248-2B epitope extends the membrane proximal target defined by previously identified bNAbs. Altogether, these data highlight the importance of the pre-fusion solvent exposed region of gp41 as a target for overlapping bNAb epitopes (Fig 6C).

### CAP248-2B is not dependent on glycans proximal to the gp120-gp41 interface for neutralization

**Fig 7:**
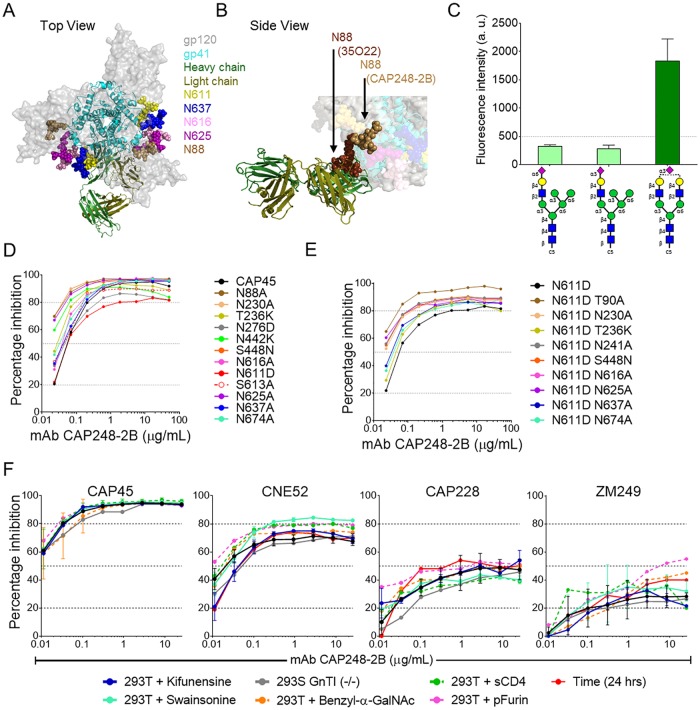
The CAP248-2B epitope is proximal to Env glycans, but not affected by glycan heterogeneity. Schematic of CAP248-2B bound trimer (colored as in Fig 4 with gp120 shown in transparent surface grey scale) including modelled (NAG)_2_MAN_3_ basic glycans at epitope proximal N88, N611, N616, N625, and N637 residues (colored brown, yellow, pink, purple, and blue respectively). Two views are shown: (A) Top view, and (B) Side view zoom. Relocation of the N88 glycan between 35O22 and CAP248-2B bound states is indicated. (C) Wong glycan array data for CAP248-2B. Weak binding was detected for two hybrid glycans, but significant binding of >500 a.u. was only detected for one biantennary mono-sialylated complex N-glycan (indicated by the glycan schema). (D) Neutralization of CAP45 by CAP248-2B when compared to epitope proximal glycan mutants. The S613A mutant that removed the glycan at N611 but maintains the amino acid side chain properties is shown with red dashed lines and open circles. Percentage inhibition was plotted on the y-axis versus CAP248-2B antibody concentration on the x-axis. (E) Neutralization of the CAP45 N611D single mutant, and various N611D including double glycan mutants, plotted as in D. (F) Neutralization of heterologous tier-2 strains CAP45, CNE52, CAP228, and ZM249 when grown normally (black lines), with co-transfected furin (dashed pink lines), or in the presence of kifunensine (blue lines), swainsonine (cyan lines), and an O-linked glycosylation inhibitor (dashed orange lines), or in a GnTI deficient cell line (grey lines). Neutralization was also assessed in the presence of sCD4 at predetermined IC_40_ concentrations for each virus (dashed green lines), or after a 24 hour virus-target cell incubation period (red lines). Graphs are plotted as in D.

With the exception of 3BC315, bNAbs identified to date targeting the gp120-gp41-gp41 interfaces depend on various highly conserved glycosylation sites for neutralization. Docking of the CAP248-2B Fab into the EM 3D reconstruction showed that CAP248-2B does not use long CDRs to penetrate the glycan shield, but instead glycans proximal to the epitope (particularly N88 and N611) need to shift to facilitate CAP248-2B binding (Fig 7A). In this model, the 35O22 bound orientation of the N88 glycan was incompatible with CAP248-2B binding, suggesting that similarly to 3BC315 [48], the N88 glycan must first be relocated (Fig 7B). Given the close proximity of the CAP248-2B epitope to several N-linked glycans in Env, particularly N88, N611, N625, and N637, we assessed whether CAP248-2B binds one/more glycans as part of its epitope. Glycan binding arrays showed that CAP248-2B only bound significantly to one of 230 glycans tested (with fluorescence intensity of greater than 500 a. u.), a biantennary mono-sialylated complex N-glycan (Fig 7C). This differs from the tri- and tetra- antennary complex glycans required by the PGT151 lineage [29]. No binding was detected to high mannose glycan. Both N88 and N611 exist predominantly as biantennary complex type glycans on BG505 SOSIP trimers [56], making these glycans candidates for CAP248-2B binding.

To assess whether glycans in gp120 or gp41 that were proximal to the CAP248 epitope were required for CAP248-2B neutralization, knock-out mutants were generated in CAP45 and tested for altered sensitivity to CAP248-2B, 8ANC195, 35O22, PGT151, and 3BC315 (Fig 7D and S6A Fig). As expected, 8ANC195 neutralization was abrogated by T236K and N276A mutations but enhanced by an N230A mutation, 35O22 neutralization was reduced by N88A, N230A, T236K, and N625A mutations, and PGT151 was negatively affected by N611D and N637A mutations (S6A Fig) [29, 44, 57]. However unlike these three bNAbs, but similar to 3BC315, CAP248-2B neutralization titres were not negatively affected by any of these glycan deleting mutations (Fig 7D).

The N276D and N611D changes resulted in slightly lower CAP248-2B neutralization plateaus, but did not affect CAP248-2B IC_50_. To test whether CAP248-2B neutralization was dependent on the N611 glycan, an S613A mutation (that also removes the N611 glycan) was introduced into CAP45. While similarly resistant to PGT151 (when compared to N611D), the S613A mutation had no effect on CAP248-2B neutralization, suggesting that unlike PGT151, CAP248-2B does not interact with the N611 glycan but rather with the amino acid side chain at position 611 (Fig 7D and S6A Fig—dashed red line). In contrast to N611, the N88A, N230A, and N625A glycan mutants did not affect neutralization plateaus, but were more potently neutralized by CAP248-2B at IC_50_ than the wild-type CAP45, supporting the observation that these glycans obscure the CAP248-2B epitope. The N88A glycan mutant was also more sensitive to 3BC315 neutralization, consistent with published data showing that this glycan partially occludes the 3BC315 epitope [48]. These data suggest that CAP248-2B was not critically dependent on any single glycan in the gp120-gp41 interface for neutralization, despite the ability to bind a complex glycan.

PGT151 is only partially affected by the individual N611D/S613A or N637A mutations, and simultaneous mutation at both N611 and N637 glycan sites is required to completely abrogate neutralization [29]. To test whether the removal of an additional glycan, in conjunction with the N611D mutation, was similarly required to knock out CAP248-2B activity, each of the glycan mutants was also made in the N611D mutant backbone (Fig 7E and S6B Fig). None of these double glycan mutant pseudoviruses showed increased resistance to CAP248-2B, though all showed partially elevated plateaus relative to the N611D mutant for CAP248-2B, with a T90A mutation (that removes the N88 glycan) having the greatest effect. Altogether these data suggest that CAP248-2B can bind to a complex glycan, perhaps at position N88 or N611, but this binding is not required for neutralization.

### CAP248-2B neutralization plateaus could not be explained by glycan heterogeneity, cleavage, or soluble CD4

While CAP248-2B did not appear to be critically dependent on glycans surrounding the gp120-gp41 interface, incomplete inhibition by HIV-1 bNAbs is often attributed to heterogeneous glycosylation within or proximal to bNAb epitopes [33]. To test whether CAP248-2B had a preference for particular N-linked glycoforms of Env, we evaluated CAP248-2B neutralization of pseudoviruses grown in the presence of N-glycosylation pathway inhibitors kifunensine or swainsonine, or grown in a GnTI deficient cell line (Fig 7F). Four sensitive strains representing different neutralization maxima (CAP45, CNE52, CAP228, and ZM249 from Fig 1D) were used. Except for the slightly enhanced sensitivity of swainsonine-grown CNE52 to CAP248-2B, there was no substantial change in the CAP248-2B neutralization plateaus when enriching for high mannose (kifunensine), medium-high mannose (GnTI (-/-)) or pre-complex only (swainsonine) glycans (Fig 7F). Therefore, the unusually low neutralization plateaus of CAP248-2B could not be completely explained by overall envelope N-linked glycan heterogeneity.

Other factors influencing intra-strain heterogeneity could potentially include O-linked glycosylation of Env, inefficient gp160 cleavage, or an exclusive preference for a CD4 activated form of envelope. While the potential role of O-linked glycosylation in envelope heterogeneity has not been extensively characterized, recombinant gp120 expressed as a monomer can be O-glycosylated in the C terminus at position T499, and at least some forms of gp160 might be O-glycosylated at position T606 in gp41 [58–62]. Both of these sites were proximal to the CAP248-2B epitope, but manipulating Env O-linked glycosylation pathways by growing pseudoviruses in the presence of benzyl 2-acetamido-2-deoxy-α-D-galactopyranoside (a modulator of mucin-like O-linked glycosylation pathways, preventing N-acetyl glucosamine addition) did not affect CAP248-2B maximum inhibition plateaus (Fig 7F—orange dashed curves). Similarly, increasing gp160 cleavage efficacy by co-transfecting pEnv and pFurin during pseudovirus production (Fig 7F—pink dashed curves), increasing the sampling of a CD4-bound conformation during the neutralization assays by pre-incubating pseudovirions with soluble CD4 at a previously determined IC_40_ value for 30 minutes (Fig 7F—green dashed curves), or increasing the incubation time between virus and antibody from 1 hour to 24 hours to increase the sampling of less frequent Env conformations (Fig 7F—red curves), did not substantially affect CAP248-2B neutralization plateaus. These data suggest that gp160 cleavage, sampling of non-native pre-fusion conformations, or O-linked glycan processing also do not contribute to the low neutralization plateaus of CAP248-2B.

### Fine mapping of the CAP248-2B epitope reveals a distinct neutralization target

**Fig 8:**
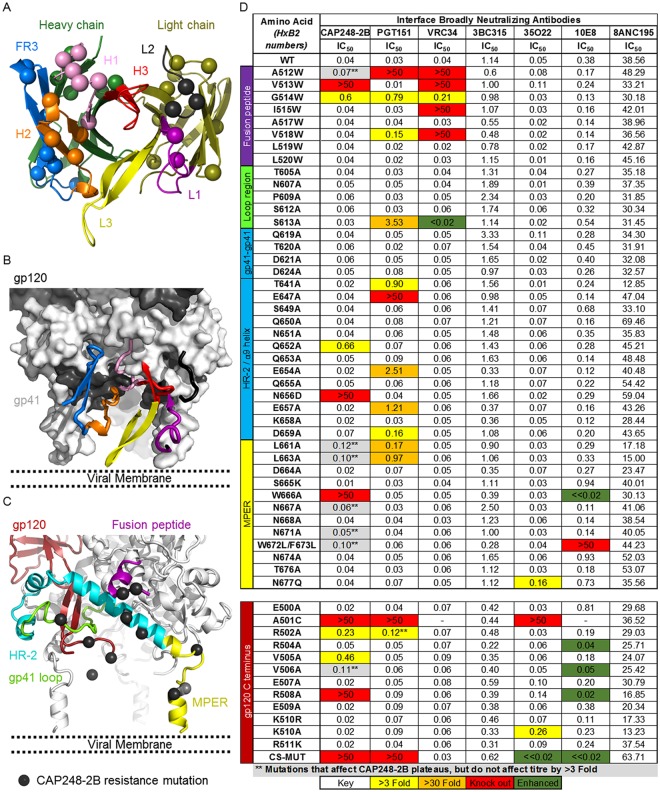
Fine mapping of the CAP248-2B epitope. (A) Cartoon of the CAP248-2B paratope (shown as a mirror image of the docked model in B, and colored as in Fig 3), showing amino acids that have affinity matured relative to the predicted germline genes with main chain Cα spheres. (B) A surface view schematic of gp41 (dark grey) and proximal regions in gp120 (light grey) showing the predicted location of CAP248-2B CDRs and FW3, coloured as in A. The approximate location of the viral membrane is indicated. (C) The envelope trimer is shown in cartoon view with the viral membrane estimated as in B. Regions of Env predicted to form part of the CAP248-2B epitope are coloured and labelled. Point mutants shown to significantly affect CAP248-2B neutralization are shown with black spheres. (D) Table showing neutralization IC_50_ titers for CAP248-2B, PGT151, VRC34, 3BC315, 35O22, 10E8, and 8ANC195 against CAP45 and various mutants. The location of each mutant in either gp41 or the gp120 C terminus is shown on the left and coloured as in C. Fold effects on IC_50_ are colored, with warmer colours to indicated increasingly negative effect.

Most of the CAP248-2B affinity maturation occurred in the heavy chain CDRs and FR3, with very limited maturation occurring in the CDR-L1 (Figs 3A and 8A). In accordance with these data, docking CAP248-2B onto an Env model that includes the MPER suggests that most of the protein-protein interactions are made by the heavy chain CDRs as well as the heavy chain FR3 (Fig 8B). There were some predicted peptide interactions for the CDR-L1 and L2 near the gp41-gp41 interface, and the CAP248-2B CDR-L3 was of the correct length to traverse the gap between the α9 helix of gp41 and the viral membrane (Fig 8B and 8C). To characterize the CAP248 epitope in finer detail, we made mutants in the gp120 C terminus (the location of escape mutations) and proximal regions of gp41 (the fusion peptide, C-C loop region, HR-2 / α9 helix, and MPER) and compared neutralization of CAP248-2B to gp41-directed bNAbs (Fig 8C and 8D).

None of the mutations universally abrogated neutralization of all the bNAbs tested, suggesting that trimer conformation was not compromised, however the A501C mutation negatively affected the neutralization of PGT151, CAP248-2B, and 35O22. Mutations in the fusion peptide at positions 513 and 514 negatively affected CAP248-2B neutralization, similar to bNAbs PGT151 and VRC34. However unlike these latter antibodies, mutations in the membrane proximal face of the α9 helix of gp41 at position Q652 substantially affected CAP248-2B neutralization, while N656D completely abrogated its activity. These sites are close enough to interact with CDR-H1 residues Glu^32^/Asp^33^ that were shown above to be critical to the CAP248-2B interaction with SOSIP trimer (Figs 5C, 5D and 8D). Mutations between positions 640–660 in the membrane distal face of the α9-helix of gp41 affected PGT151 neutralization, consistent with its epitope. Lastly, mutations in the MPER at position 666 abrogated CAP248-2B neutralization, similar to MPER antibody 2F5, and mutations at 672/673 also substantially affected neutralization, similar to 10E8/4E10. Several of the mutations in the fusion peptide, MPER, and gp120 C terminus appeared to affect CAP248-2B maximum inhibition percentages, but did not affect CAP248-2B IC_50_ values more than three-fold.

Mutations in the gp120 C terminus affected both CAP248-2B and PGT151 neutralization, and despite observations that PGT151 does not bind the gp120 C terminus directly (its access is occluded by gp41) the CAP45(CS-Mut) virus, that is completely resistant to CAP248-2B, was also resistant at IC_50_ to PGT151 (neutralization plateaus just under 50%). Conversely, 35O22 and 10E8 neutralization of CAP45(CS-Mut) was considerably more potent than the wild-type CAP45 virus. All of the mutations affecting CAP248-2B overlapped with the predicted antibody binding footprint, confirming their importance in the CAP248-2B epitope (Fig 8C—black spheres). Altogether, these data confirm the overlap between the CAP248-2B epitope and the epitopes for PGT151, VRC34, and 10E8, but also highlight the distinct nature of the CAP248 plasma bNAb epitope which includes the membrane proximal half of the α9 helix in gp41, and the gp120 C terminus.

### Escape from CAP248-2B exposes proximal epitopes for anti-gp41 broadly neutralizing antibodies

**Fig 9:**
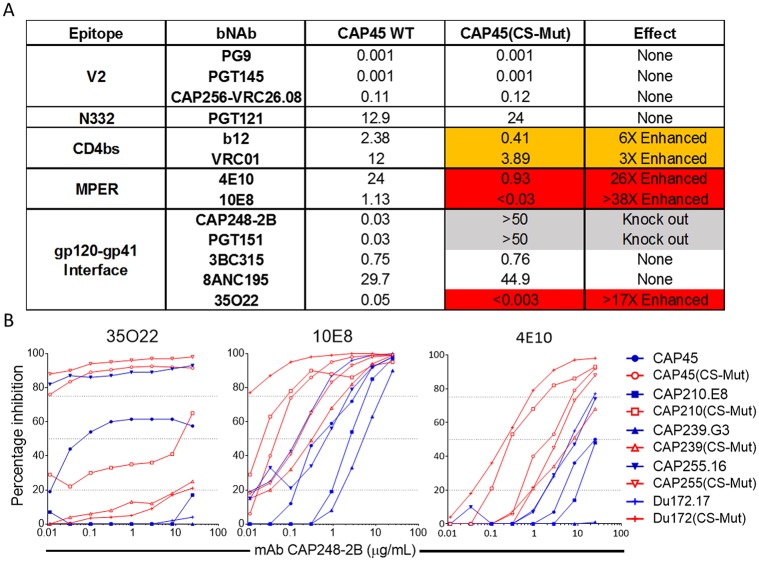
Escape mutations from CAP248-2B enhance the neutralization of broadly neutralizing antibodies that bind to gp41. (A) Neutralization of CAP45 and a mutant variant that includes the six gp120 C-terminal mutations identified in CAP248 autologous sequences (CS-Mut), by broadly neutralizing antibodies with epitopes in V2, V3, the CD4bs, the MPER, and the gp120-gp41 interface. Fold changes less than three are within the variation of the assay (no effect). Fold enhancement in neutralization sensitivity to CAP248-2B is indicated in orange (3–10 fold increased sensitivity) and red (> 10 fold increased sensitivity). Conferred resistance to the mutant virus at IC_50_ is shown by grey shading. (B) Neutralization of five paired WT (blue) and CS-Mut (red) viruses by 35O22, 10E8, and 4E10.

The enhancement of 35O22 neutralization after the introduction of cleavage site mutations into CAP45 suggested a role for the gp120 C terminus in Env conformation. To investigate this, the neutralization of CAP45(CS-Mut) was compared to wildtype CAP45 for bNAbs with diverse epitopes on HIV-1 Env (Fig 9A). CAP45(CS-Mut) neutralization by V2 and N332 antibodies remained unchanged relative to wildtype. The neutralization of CD4bs antibodies VRC01 and b12 was marginally enhanced, but neutralization of MPER bNAbs 4E10 and 10E8 was enhanced by 26 and 38 fold respectively. CAP45 is resistant to 2F5 and Z13e1, so these bNAbs were not tested. To determine whether this effect was specific to CAP45, the six CAP248 autologous escape mutations (CS-Mut) were simultaneously introduced into four additional heterologous pseudoviruses. Similar to CAP45, all four of these mutant pseudoviruses were between 10 and 100 fold more sensitive to 35O22, 4E10, and 10E8 neutralization (Fig 9B). In some instances introduction of the CS-mutations conferred sensitivity to 35O22 or 4E10, where the wild-type virus was completely resistant at the concentrations tested. These data show that mutations in the C terminus of gp120 induce localised effects in trimer conformation that specifically enhance sensitivity to MPER bNAbs.

## Discussion

The identification of HIV-1 bNAbs with overlapping epitopes in the gp120-gp41 interface has greatly expanded our knowledge of the regions of the envelope trimer susceptible to neutralization [29, 44, 47, 48, 63–65]. Here, we isolated a monoclonal antibody called CAP248-2B that targeted a membrane proximal epitope in the gp120 C terminus-gp41 and gp41-gp41 interfaces. This epitope overlapped with, but was distinct from the epitopes for bNAbs PGT151, VRC34, 35O22, 3BC315, and 10E8. Together with 8ANC195, these epitopes surround the base of the HIV-1 envelope trimer, forming a continuum of neutralization vulnerability (Fig 6C) [49]. Unlike many other bNAbs, CAP248-2B neutralization plateaus could not be completely explained by glycan heterogeneity. Furthermore, rare mutations in the gp120 C terminus that mediated escape from CAP248-2B increased the sensitivity of HIV-1 viruses to MPER antibodies. Thus the identification of new antibodies targeting this supersite of vulnerability may provide important insights for vaccine design.

CAP248-2B was isolated from a CAPRISA donor (CAP248) who showed plasma neutralization breadth of nearly 60% against a multi-subtype panel and 80% against subtype C viruses. While CAP248-2B did not recapitulate the donor’s plasma breadth at IC_50_, the neutralization profile at IC_20_ strongly suggested that this lineage was responsible for CAP248 broad neutralization. The CAP248-2B epitope was structurally proximal to glycans at N88, N230, N611, and N625, and was able to bind to a biantennary complex glycan, but CAP248-2B neutralization was not dependent on any single glycan, or any double glycan knock-outs that included N611D. It is possible that other members of this antibody lineage in CAP248 plasma have matured to become glycan dependent, however the ability to bind glycans that are not critical for neutralization has also been described for other bNAbs [66, 67]. These data would support recent evidence for the common occurrence of neutralization plateaus across all bNAb classes, including those that do not require glycan for effective neutralization [33, 34]. Potential mechanisms for incomplete neutralization were explored, such as the rate of furin cleavage or random sampling of CD4-induced transition intermediates, but did not affect the low plateaus for CAP248-2B. It is possible that CAP248-2B neutralization plateaus were the result of varied accessibility of the gp120 C terminus, unfavourable binding kinetics (eg: varied off-rates between various strains), or a preference for different glycoforms at two or more N-linked glycan sites. This could explain why shifting the overall glycosylation profile of a viral strain in one direction (e.g. by growing a pseudovirus in the presence of a glycosylation pathway inhibitor) had no substantial effect on the neutralization plateau. Alternatively, our data suggest an additional contributor to envelope heterogeneity, which could be an important confounder for vaccine immunogen design.

Structural analysis indicated that the CAP248-2B paratope was conformationally variable. Divergent CDR-H3 conformations that were only seen because of crystal packing may represent a level of plasticity that could have evolved to better accommodate Env sequence variation. Many bNAb epitopes are composed of structurally dynamic components, such as lipid membranes or large glycan moieties. For instance, the N88 glycan is oriented towards the viral membrane when bound by 35O22, but shifts into an orientation that would clash with 35O22 binding to allow for 3BC315 to access its epitope [48, 65]. As a result, bNAb affinity maturation often rigidifies the paratope by hydrogen bonding and/or disulphide bonds within the CDRs. This optimization of the “lock-and-key” fit between antigen and antibody can result in increased potency by reductions in binding entropy. Conversely the additional flexibility observed for CAP248-2B may contribute to its low potency.

The unusually long CDR-L3 of CAP248-2B was specifically angled towards the viral membrane but its tip retains a level of plasticity that may assist in interacting with dynamic viral lipids. In this way, the CAP248-2B light chain CDR-L3 performs a similar function to the heavy chain CDR-H3 of MPER targeting bNAbs 2F5, Z13e1, 4E10, and 10E8 which all extend hydrophobic residues at the CDR-H3 loop tip to anchor the antibody in the viral membrane. While 2F5 and 4E10 are significantly autoreactive, we saw no evidence of this for CAP248-2B, similar to 10E8 and 35O22. In addition to sharing a common mechanism of lipid recognition, CAP248-2B, 35O22, and MPER bNAbs all approach the HIV-1 trimer very close to the viral membrane. These antibodies may require the trimer to alter its orientation or position relative to the viral membrane for them to access their epitopes [44]. As a consequence of this epitope occlusion, MPER bNAbs may not bind as well to pre-fusion native trimers [68]. From our EM docking analyses, it appears that CAP248-2B recognizes the pre-fusion “closed” state of the HIV-1 envelope trimer. It remains to be determined what structural rearrangements (if any) in the trimer are required for CAP248-2B to properly access its epitope.

In accordance with its low angle of binding, escape from CAP248-2B occurred in both the gp160 cleavage motifs. Of the six identified mutations, only V505M individually affected CAP248-2B neutralization. In autologous viruses, mutations at positions 500, 502, 507, 508, and 509 all occurred by two years post-infection, while variants at position 505 were only detectable after three years of infection. Based on these kinetics, it is likely that mutations at positions 500, 502, 507, 508, and 509 accumulated first in response to earlier members of the CAP248 bNAb lineage, with the eventual selection of extremely rare mutations at position 505 by later members of the antibody lineage. It is also possible that the other mutations affect local Env conformation, conferring escape through an indirect mechanism. Similarly, we identified a cluster of autologous gp41 mutations overlapping the CAP248-2B epitope that may have played a role in escaping earlier lineage members. Future experiments to isolate these early members of the lineage will help to understand how virus-antibody co-evolution led to the development of CAP248-2B. In addition, the isolation of more potent variants of CAP248-2B should help to explain the mechanisms of incomplete neutralization for this new interface targeting antibody.

In addition to mediating escape from CAP248-2B, mutations in the gp120 C terminus conferred partial resistance to PGT151. Based on EM 3D reconstructions PGT151 does not bind the gp120 C terminus [63], suggesting that these mutations have the ability to affect envelope conformation in a way that confers resistance to PGT151. Conversely, the gp120 C-terminal mutations also had the unexpected effect of dramatically enhancing the neutralization of 35O22 (targeting a membrane proximal epitope) and bNAbs targeting the MPER, suggesting that these conformational effects may assist in raising gp41 relative to the membrane [44, 69–72], perhaps by increasing the frequency at which membrane associated Env trimers sample a CD4-induced conformation, without first having to engage the CD4 receptor. Importantly, this effect did not make viruses globally sensitive to HIV-1 antibodies, but was specific for bNAbs with membrane proximal epitopes such as 4E10, 10E8, and 35O22. Incorporating these mutations into membrane bound HIV-1 trimer immunogens may therefore improve the antigenicity of bNAb epitopes in gp41.

Overall these data expand the recently identified gp120-gp41 interface supersite to include the gp120 C terminus, highlighting the importance of this region as a vaccine target. This region of Env was also the target for neutralizing antibodies elicited in rabbits by SOSIP trimer immunogens [73]. Further characterization of the CAP248 bNAb epitope could therefore inform pathways through which these sorts of antibodies might achieve neutralization breadth. Future experiments should also aim to determine whether membrane-bound CS-Mut trimers successfully engage MPER bNAb precursors, thus overcoming an important barrier to the induction of MPER bNAbs. Furthermore, defining additional glycan independent mechanisms of envelope heterogeneity will have implications for the use of bNAbs in both passive and active immunization strategies.

## Materials and Methods

### Ethics statement

The CAPRISA Acute Infection study in adult women received ethical approval from the Universities of KwaZulu-Natal (E013/04), Cape Town (025/2004), and the Witwatersrand (MM040202). CAP248 provided written informed consent for study participation.

### CAPRISA 002 Acute Infection cohort

The CAPRISA Acute Infection cohort is comprised of women at high risk of HIV-1 infection in Kwa-Zulu Natal, South Africa [15]. Blood samples collected at regular intervals from seroconversion through to the initiation of antiretroviral therapy were cryopreserved as individually processed PBMC, serum and plasma samples.

### B cell culture

Cryopreserved CAP248 PBMC were thawed, washed and suspended in medium containing 10% foetal bovine serum (FBS) and antibiotics. B cells were enriched by negative selection with immunomagnetic beads (Miltenyi), and were cultured at 25 cells per well in Iscove’s Modified Dulbecco’s Medium (IMDM) containing 10% FBS, 2 μg/mL CpG2006, 100 units/mL rIL-2, rIL-21 (50 ng/mL) with 3T3msCD40L as feeder cells (a gift of Mark Connors) [45], plated at 2500 cells/well in multiple 96 well plates. rIL-2 was obtained from the NIH AIDS Reagent Program as provided by Maurice Gately (Hoffmann-La Roche). Fresh medium containing growth factors was added after 7 days of culture and after each antibody screening procedure. B cell culture fluids were screened from day 14 for neutralizing activity against CAP45 pseudovirus in an adaptation of the single-cycle TZM-bl neutralization assay as previously described [74].

### Cloning and expression of human immunoglobulin genes

B cells from wells testing positive for antibody were stored in RNAlater (Ambion). VH, Vκ, or Vλ genes were amplified in separate reactions from RNA using a one-step RT-PCR (Invitrogen SuperScript III kit with Platinum Taq High Fidelity polymerase) with previously described primer mixes [75]. For expression vector assembly, forward primers included a 25 nucleotide non-annealing 5’ tag sequence, which was homologous to the immunoglobulin leader sequence at the 3’ end of the CMV promoter fragment. Reverse primers were designed to overlap the 5’ end of the immunoglobulin constant region for each vector. Linear expression constructs were assembled by overlapping PCR between two DNA fragments containing the CMV promoter and immunoglobulin leader sequence or the constant region sequences for IgG1, kappa or lambda genes followed by a C-terminal BGH poly A sequence. These were co-transfected into 293T cells, and supernatant fluids were tested for neutralization activity. This step allowed rapid detection of pairs of VH and VL chain genes that functioned together to produce antibody. To produce monoclonal antibodies, the In-Fusion cloning system (Clontech) was used to insert re-amplified pairs of VH and VL gene fragments into pLM2 expression plasmids similar to previously described [76], but modified to contain the immunoglobulin leader sequence in the linear vectors. Expression plasmids were linearized by restriction enzymes acting on sites within the multiple cloning site of plasmid (EcoRI for IgG1 and lambda, BsiWI for kappa). Linearized vectors were then PCR amplified with primer pairs designed to create terminal sequences that were homologous to 5’ and 3’ terminal sequences of the variable region insert fragments, allowing insertion of the VH and VL fragments into linearized plasmids by the activity of the In-Fusion enzyme as described [77]. The resulting plasmids were transformed in JM109 cells. A previously described strategy was used to identify the correct pair of VH and VL clones responsible for antibody production [78]. Subsequent sequencing of multiple clones showed that only one heavy and one light chain were capable of directing mAb synthesis.

### Cell lines

CD4^+^/CCR5^+^ TZM-bl HeLa cells were obtained from the NIH AIDS Research and Reference Reagent Program, Division of AIDS, NIAID, NIH (developed by Dr. John C. Kappes, and Dr. Xiaoyun Wu [79,80]). 293T cells were obtained from Dr George Shaw (University of Alabama, Birmingham, AL). Adherent cell lines were cultured at 37°C, 5% CO2, in DMEM containing 10% heat-inactivated fetal bovine serum (Gibco BRL Life Technologies) and supplemented with 50 ug/ml gentamicin (Sigma). Cells were routinely disrupted at confluency with 0.25% trypsin in 1 mM EDTA (Sigma) every 48–72 hours. 293F suspension cells were cultured in 293Freestlye media (Gibco BRL Life Technologies) at 37°C, 10% CO2, 125RPM and diluted twice a week to between 0.2 and 0.5 million cells/mL.

### Single genome amplification

The single genome amplification of HIV Env has been previously described [79]. Briefly, CAP248 viral RNA was isolated using the Viral RNA Extraction Kit (QIAGEN), to serve as a template for Superscript III Reverse Transcriptase (Invitrogen) in cDNA generation. Residual RNA was degraded with RNaseH (Invitrogen) and CAP248 envelope genes were amplified by a nested PCR approach using Platinum Taq (Invitrogen). PCR products were cleaned up (QIAGEN) and sequenced with the ABI Prism Big Dye Terminator Cycle Sequencing Ready Reaction kit (Applied Biosystems) on the ABI 3100 automated genetic analyser, assembled using Sequencher v.4.5 (Genecodes), and compiled into working alignments in Bioedit v.7.0.5.3.

### Pseudovirus production and site-directed mutagenesis

Plasmids expressing the HIV Env of interest were co-transfected with pSG3DEnv backbone expressing plasmids (obtained from the NIH AIDS Research and Reference Reagent Program, Division of AIDS, NIAID, NIH) into 293T cells using PEI-MAX 40,000 (Polysciences). Cultures were incubated for 48 hours at 37°C, then filtered through 0.45 μm and frozen in DMEM, 20% FBS to yield Env-pseudotyped viruses capable of a single round of infection only. Mutant envelope genes were generated with the QuikChange Lightning Kit (Stratagene), confirmed by DNA sequencing, and transfected as above. For the glycan heterogeneity experiments, pseudoviruses were grown as above in the presence of 25M glycosylation inhibitor, or in 293S GnTI(-/-) cells.

### Neutralization assays

Neutralization assays were performed in TZM-bl cells as previously described [12, 80]. Neutralization is measured as a reduction in relative light units after a single round of pseudovirus infection in the presence of the monoclonal antibody or plasma sample of interest. Samples were serially diluted 1:3 and the ID_50_/IC_50_ calculated as the dilution at which the infection was reduced by 50%.

### Protein production

For CAP248-2B antibody expression, plasmids separately encoding heavy and light chain genes were co-transfected into 293F cells with PEI-MAX 40,000 (Polysciences). To make CAP248-2B Fab, the HRV-3C protein cleavage site (GLEVLFQGP) was introduced into the heavy chain gene between Fab and Fc fragments by PCR. Expressed full length mAb was digested with HRV-3C enzyme (Merck Millipore) at 25°C for four hours, and then the separated Fab fragments were purified by sequential negative selection over protein A, and positive selection by gel filtration using a superdex 200 (GE Healthcare). Cells were cultured for seven days in 293Freestyle media at 37°C, 10% CO_2_, then harvested supernatants were 0.22 μm filtered and purified using protein A. Trimers were expressed previously described [65], and purified from 0.22 μm filtered supernatants with an Ni-NTA column (30mM Imidazole wash and 400mM Imidazole elution buffers at pH7), and then by CAP248-2B mAb bound to protein A. The Fab-trimer complexes were eluted by digestion with HRV-3C (which also removed the His6 tag from gp41), and further purified by gel filtration using a superdex 200 column (GE Healthcare).

### Enzyme-Linked Immunosorbent Assay (ELISA)

HisTagged trimers were coated at 2 μg/mL in PBS onto nickel coated 96 well plates (Thermo) for one hour at 25°C. Plates were washed and then probed with serial dilutions of HIV-1 monoclonal antibody for one hour at 25°C. This process was repeated using an anti-Fc/HRP conjugate to detect trimer-bound antibodies. Antigen-antibody complexes were detected by incubating with 100 μL of enzyme substrate for five minutes and then the reaction was stopped with 25 μL of 1 M HCl. Absorbance was read at 450 nm.

### Protein X-ray crystallography

Concentrated aliquots were stored at 4°C. 576 crystallization conditions were screened in 96 well plates (Corning) using the Cartesian Honeybee crystallization robot by sitting drop vapour diffusion in 400 nL drops at 25°C containing 50% mother liquor. Crystal hits were hand-optimised in 15 well hanging drop diffusion plates at 25°C in 1 μL drops containing 50% mother liquor. All crystallographic diffraction data was collected at the Advanced Photon Source (Argonne National Laboratory) SER-CAT ID-22 beamline, at a wavelength of 1.00 Å, 100K, and processed with HKL2000. Model building and refinement was handled with COOT v0.8 and PHENIX v1.9–1692 software packages respectively, using 5% of the data as an R-free cross validation test set, and hydrogens were refined to minimise clashes. The unliganded CAP248-2B Fab was first crystallized in 10.25% PEG4000, 87.5 mM ammonium sulphate, and flash frozen in 25% PEG400 as a cryoprotectant. This crystal diffracted to a resolution of 2 Å (PDB-ID: 5MP6) and phasing by molecular replacement was done using PDB-IDs: 4QHK and 3B2U as search models. We could not reliably build the constant domain for one of the two Fabs in the asymmetric unit, which appeared to have considerable mobility within the crystal lattice, resulting in poor RSRZ scores for regions of these chains. This first structure served as the search model for the second crystal structure obtained in 7.5% PEG4000, 12.5% isopropanol, 0.1 M sodium citrate (pH5.6), and flash frozen in 30% ethylene glycol as a cryoprotectant, which diffracted with I/αI >2 to a resolution of 3.1 Å, with data up to 2.8 Å (PDB-ID: 5F89). All structural images were generated in PyMOL Molecular Graphics System, Version 1.3r1edu, Schrodinger LLC., or UCSF Chimera [81].

### Negative stain Electron Microscopy (EM)

BG505-CAP45 SOSIP trimers were incubated with a 6 molar excess of CAP248-2B Fab overnight at room temperature and the complexes were diluted to ~0.03 mg/mL in Tris-buffered saline prior to application onto a carbon-coated 400 Cu mesh grid (Electron Microscopy Sciences) that had been glow discharged at 20 mA for 30 seconds. The grids were stained with 2% (w/v) NanoW (Nanoprobes) for 7 s, blotted, and stained for an additional 15 s. Samples were imaged on an FEI T12 electron microscope operating at 120 keV, with an electron dose of ~25 electrons/Å^2^ and a magnification of 52,000x that resulted in a pixel size of 2.05 Å at the specimen plane. Images were acquired with Leginon [82], using a Tietz TemCam F416 camera and a nominal defocus range of 1000–1500 nm. Stage tilts between -50° and 0° using 10° increments were performed to increase the amount of unique views to aid with 3D reconstruction. Automated particle picking, stack creation, and initial 2D classification were performed in the Appion software suite [83]. Classes representing noisy alignments, neighboring particles, unbound Fab, or ligand-free trimers were discarded and representative class averages with unique views of the SOSIP-CAP248-2B complex were used to generate an initial common-lines model using EMAN2 [84], followed by refinement against all 28,215 particles in Sparx [85], with C3-symmetry imposed. The resolution of the final reconstruction is ~20 Å based on a Fourier shell correlation of 0.5. Two-dimensional back projections of the final 3D models were generated using EMAN [84].

### Autoreactivity assays

Antibody binding to cardiolipin, or reactivity with Hep-2 epithelial cells (ZEUS Scientific) was assessed as previously described [35] per the manufacturer’s protocol. Antibodies were scored as positive or negative at 50 μg/mL when compared to a no antibody control. The monoclonal antibodies 4E10 and 35O22 were included as positive and negative controls respectively.

### Cell-surface binding assay

CAP45.2.00.G3J (Genbank: EF203960) *env* plasmid was codon optimised (GenScript) and truncated at the cytoplasmic tail to increase surface Env content [86]. Following restriction digest cloning this plasmid was transiently transfected using TrueFect-MAX (United Biosystems) into HEK/293T cells. Two days after transfection, cells were labelled with Live/Dead Fixable Aqua Dead Cell Stain (Life Technologies) followed by biotinylated CAP248-2B and serially diluted unlabelled competitor antibodies (CAP248-2B, 3BC315, 35O22, PGT151. 8ANC195, 4E10, and control antibody Palivizumab). After incubation and three washes with 5% FBS in PBS, cells were stained with Streptavidin-PE (Life Technologies) at a 1:300 dilution. Reverse competition assays were also performed with biotinylated 3BC315, 35O22, PGT151, 8ANC195 and 4E10 and serially diluted CAP248-2B or Palivizumab. Cells were analysed on a BD FACS Aria II (Becton Dickinson) and binding was measured as the median fluorescence intensity (MFI) for each sample minus the MFI of the cells stained with the detection antibody only.

### SOSIP capture assays

BG505-CAP45 chimeric SOSIP trimers (expressed without additional pFurin to reduce cleavage efficacy, and therefore containing both cleaved and uncleaved trimers) were captured from 293F supernatants by monoclonal antibodies CAP256-VRC26.09 and CAP248-2B covalently bound to protein A. Eluted proteins were assessed on SDS-PAGE with and without dithiothreitol to determine the ratio between cleaved and uncleaved gp160.

### Wong glycan binding arrays

Pure amine-functional glycans were printed onto NHS-activated glass slides, blocked with ethanolamine, and probed with CAP248-2B monoclonal antibody as described previously [29, 87]. Reactivity was calculated by the mean intensity minus the mean background, and preformed with different secondary antibodies to limit signal: noise ratio, where binding with a fluorescence intensity of greater than 500 a. u. was considered positive. No binding was detected against high-mannose glycans.

## Supporting Information

S1 Fig Conformational differences between unliganded structures of CAP248-2B explained by crystal packing. Predicted germline alleles, CDR3 lengths, and mutation frequencies for CAP248-2B from the IMGT database [88].
(TIF)

S2 Fig Comparison of IC_20_ and IC_50_ titres for weakly neutralizing HIV-1 antibodies. Three weakly neutralizing antibodies, 447-52D, 17b and HK20 were tested against a 45 virus panel. Titres at both IC_20_ and IC_50_ are shown, and coloured as in Fig 1. Percentage breadth is indicated at the bottom.
(TIF)

S3 Fig Selection pressure in conserved bNAb epitopes does not contribute to escape from CAP248-2B. (A) Alignments of five different regions in CAP248 autologous envelope sequences at nine weeks (study enrolment), 1, 2, 3 and 3.5 years post-infection, that overlap with previously described broadly neutralizing antibody epitopes in V2 (boxed in purple), C1/C2 (boxed in brown), V3 (boxed in orange), and the MPER (boxed in blue). N-linked glycosylation sequons are shaded grey. (B) CAP248-2B neutralization of the heterologous strain CAP45, compared to mutants containing potential autologous escape mutations identified from CAP248 sequences. CAP45 already had the N230 glycan, so the N230D reverse mutation was tested (grey dashed line). Percent inhibition (y-axis) is plotted against CAP248-2B concentration (x-axis). (C) CAP248-2B neutralization of CAP45 and mutants with known resistance mutations to common bNAbs (not identified in CAP248 sequences), plotted as in B. (D) Longitudinal analysis of CAP248 plasma neutralization tires at yearly intervals when measured against wild type CAP45, or the CS-Mutant variant.
(TIF)

S4 Fig Supporting crystal structure, ELISA, and negative stain EM data. (A) Cartoon representation of neighboring asymmetric units from the 2 Å resolution dataset showing crystal packing in the antibody paratope. The heavy and light chains of Fab1 in the first unit are colored forest and olive green respectively, and in the second unit dark and light blue. The CDR-H3 (red), and CDR-L3 (yellow) are indicated. (B) ELISA data comparing the binding of various HIV-1 antibodies to monomeric gp120 (top panel) and monomeric gp145 (bottom panel). Absorbance readings are plotted on the y-axis and antibody concentration on the x-axis. (C) Reference-free 2D class averages (D) 2D back-projections of the final mode (E) 3D reconstructions (top and side views) (F) Fourier Shell Correlation (FSC) curves with estimated resolution using an FSC cut-off of 0.5. Samples were stained with NanoW.
(TIF)

S5 Fig CAP248-2B blocks the binding of 35O22, 3BC315, and PGT151 to cell surface envelope trimers. (B) Binding of labelled HIV-1 bNAbs 35O22, 3BC315, PGT151, 8ANC195, and 4E10 to cell-surface anchored HIV-1 trimers by flow cytometry, in the presence of increasing concentration of unlabeled CAP248-2B. Median fluorescence intensity (MFI) is shown on the y-axis, and increasing concentrations of CAP248-2B or palivizumab are plotted on the x-axis. Decreasing MFI signals correspond to increasing competition by CAP248-2B.
(TIF)

S6 Fig Glycan dependence of known gp120-gp41 interface targeting bNAbs. (A) Neutralization of CAP45 by broadly neutralizing antibodies that target the gp120-gp41 interface, when compared to CAP248-2B epitope proximal glycan mutants. The S613A mutant that removed the glycan at N611 but maintains the amino acid side chain properties is shown with red dashed lines and open circles. Percentage inhibition is plotted on the y-axis versus CAP248-2B antibody concentration on the x-axis. (B) Neutralization of the CAP45 N611D single mutant, and various N611D including double glycan mutants, plotted as in A.
(TIF)

S1 Table Crystallographic data and refinement statistics (molecular replacement) for the antigen binding fragment of CAP248-2B. Data collection and refinement statistics for the two unliganded CAP248-2B Fab crystal structures.
(TIF)
