# Supplementary figures and images for: Structure and Recognition of a Novel HIV-1 gp120-gp41 Interface Antibody that Caused MPER Exposure through Viral Escape
Source: PLoS Pathog. 2017 Jan 11;13(1):e1006074. doi: 10.1371/journal.ppat.1006074 (PMC5226681; doi:10.1371/journal.ppat.1006074)

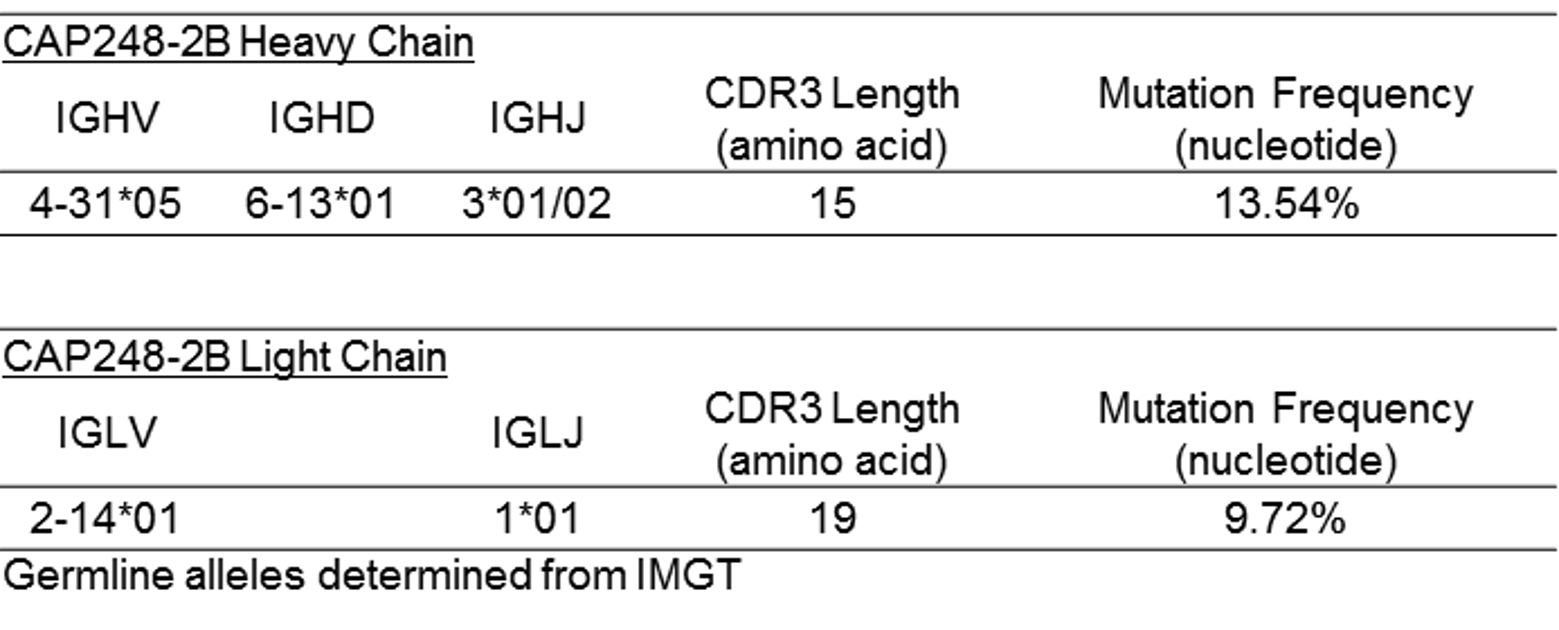

Supplement: S1 Fig — Predicted germline alleles, CDR3 lengths, and mutation frequencies for CAP248-2B from the IMGT database [88]. (TIF) [file ppat.1006074.s001.tif]

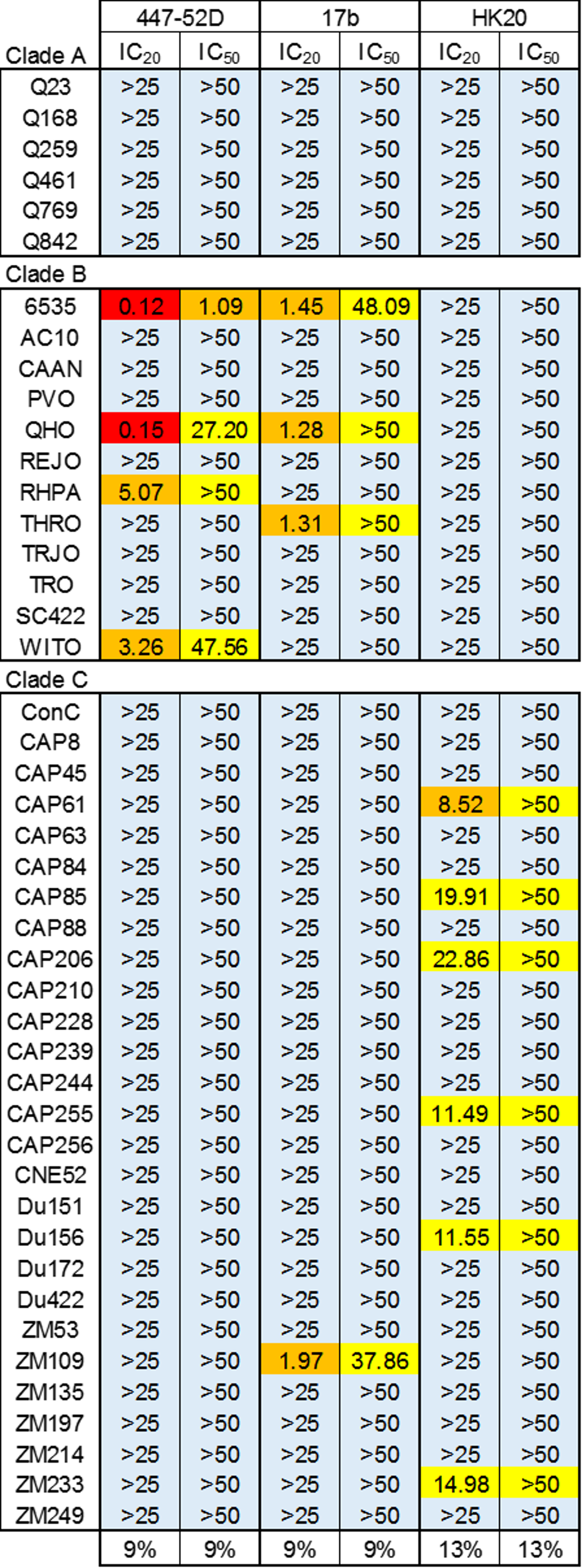

Supplement: S2 Fig — Three weakly neutralizing antibodies, 447-52D, 17b and HK20 were tested against a 45 virus panel. Titres at both IC20 and IC50 are shown, and coloured as in Fig 1. Percentage breadth is indicated at the bottom. (TIF) [file ppat.1006074.s002.tif]

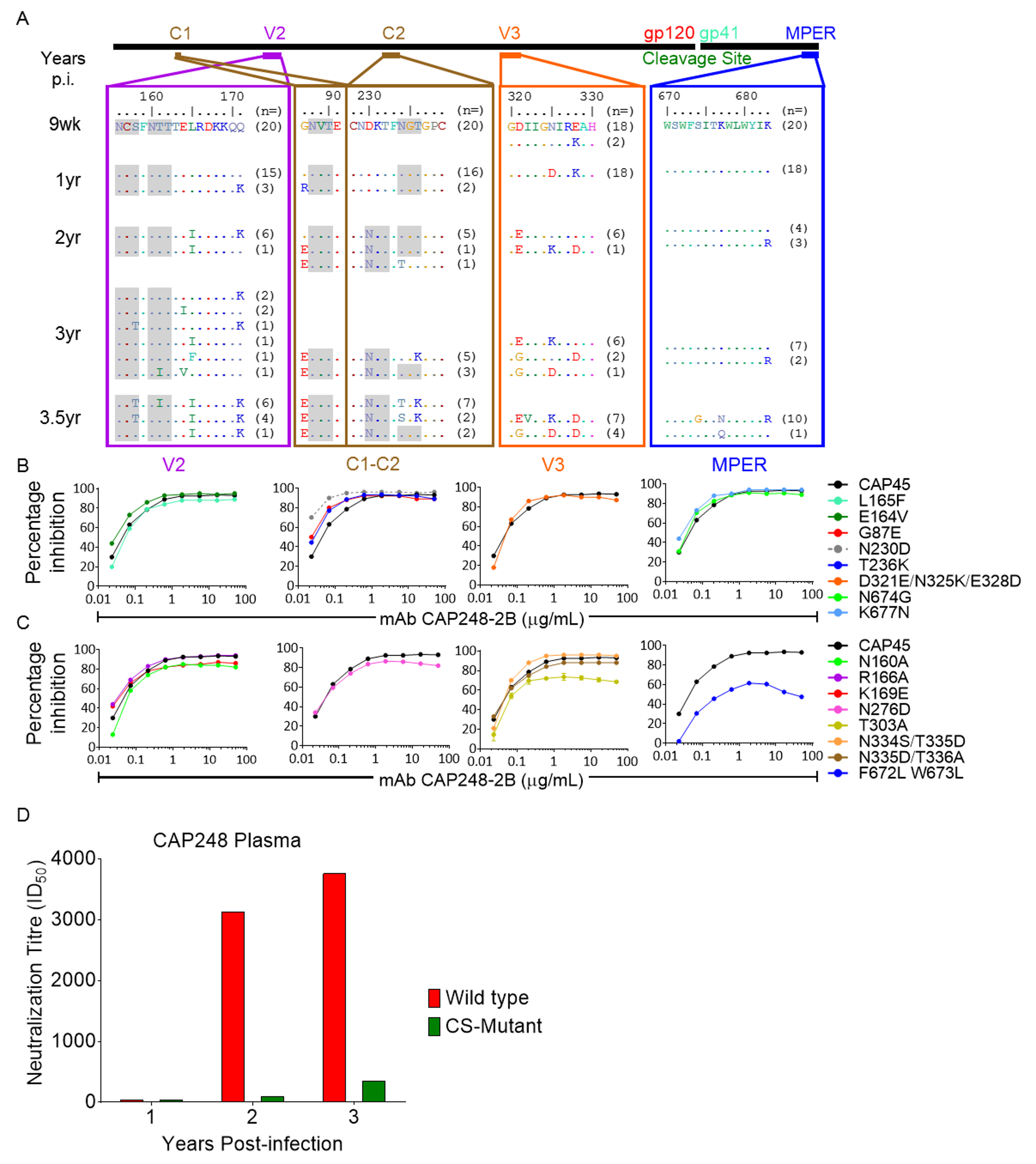

Supplement: S3 Fig — (A) Alignments of five different regions in CAP248 autologous envelope sequences at nine weeks (study enrolment), 1, 2, 3 and 3.5 years post-infection, that overlap with previously described broadly neutralizing antibody epitopes in V2 (boxed in purple), C1/C2 (boxed in brown), V3 (boxed in orange), and the MPER (boxed in blue). N-linked glycosylation sequons are shaded grey. (B) CAP248-2B neutralization of the heterologous strain CAP45, compared to mutants containing potential autologous escape mutations identified from CAP248 sequences. CAP45 already had the N230 glycan, so the N230D reverse mutation was tested (grey dashed line). Percent inhibition (y-axis) is plotted against CAP248-2B concentration (x-axis). (C) CAP248-2B neutralization of CAP45 and mutants with known resistance mutations to common bNAbs (not identified in CAP248 sequences), plotted as in B. (D) Longitudinal analysis of CAP248 plasma neutralization tires at yearly intervals when measured against wild type CAP45, or the CS-Mutant variant. (TIF) [file ppat.1006074.s003.tif]

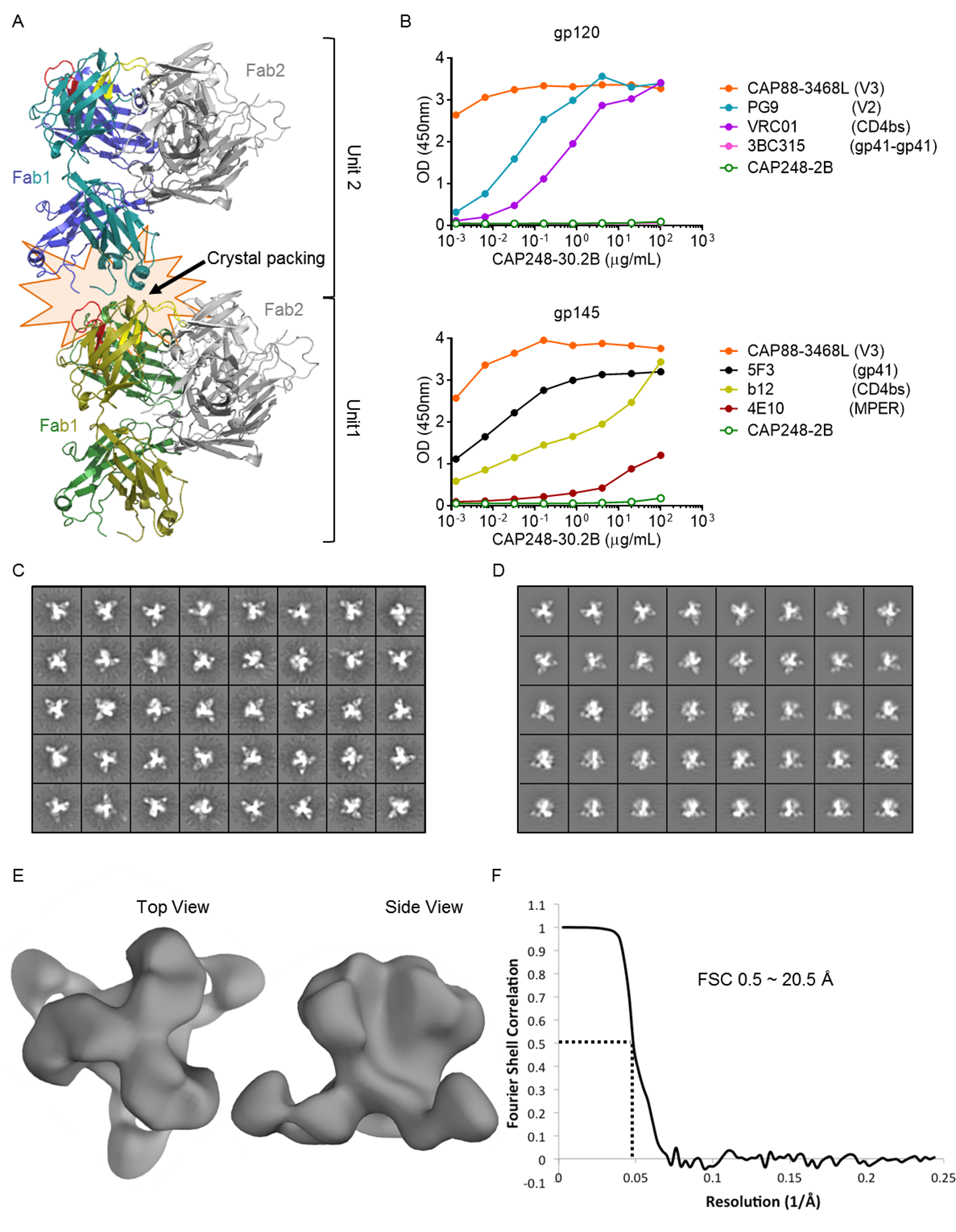

Supplement: S4 Fig — (A) Cartoon representation of neighboring asymmetric units from the 2 Å resolution dataset showing crystal packing in the antibody paratope. The heavy and light chains of Fab1 in the first unit are colored forest and olive green respectively, and in the second unit dark and light blue. The CDR-H3 (red), and CDR-L3 (yellow) are indicated. (B) ELISA data comparing the binding of various HIV-1 antibodies to monomeric gp120 (top panel) and monomeric gp145 (bottom panel). Absorbance readings are plotted on the y-axis and antibody concentration on the x-axis. (C) Reference-free 2D class averages (D) 2D back-projections of the final mode (E) 3D reconstructions (top and side views) (F) Fourier Shell Correlation (FSC) curves with estimated resolution using an FSC cut-off of 0.5. Samples were stained with NanoW. (TIF) [file ppat.1006074.s004.tif]

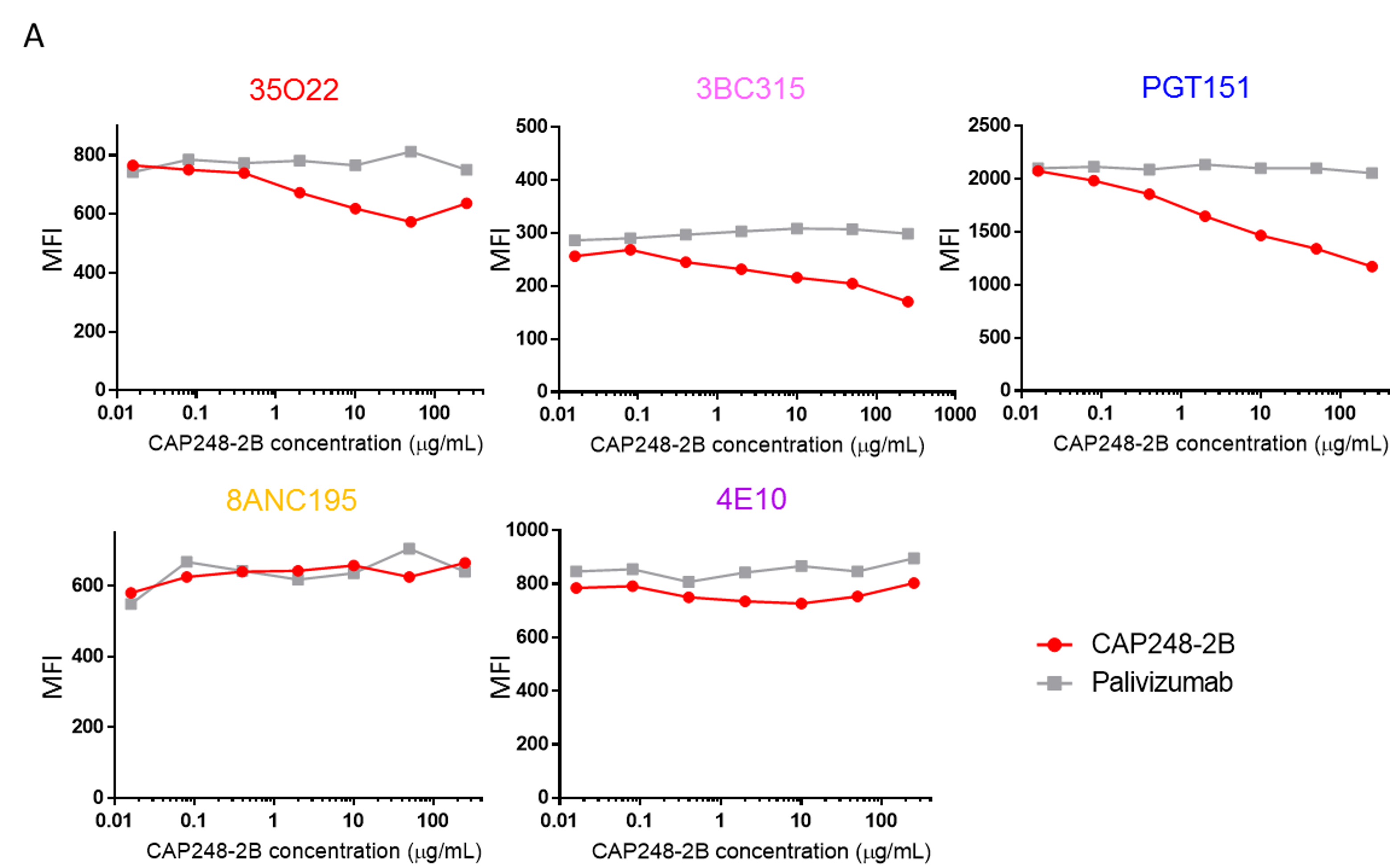

Supplement: S5 Fig — (B) Binding of labelled HIV-1 bNAbs 35O22, 3BC315, PGT151, 8ANC195, and 4E10 to cell-surface anchored HIV-1 trimers by flow cytometry, in the presence of increasing concentration of unlabeled CAP248-2B. Median fluorescence intensity (MFI) is shown on the y-axis, and increasing concentrations of CAP248-2B or palivizumab are plotted on the x-axis. Decreasing MFI signals correspond to increasing competition by CAP248-2B. (TIF) [file ppat.1006074.s005.tif]

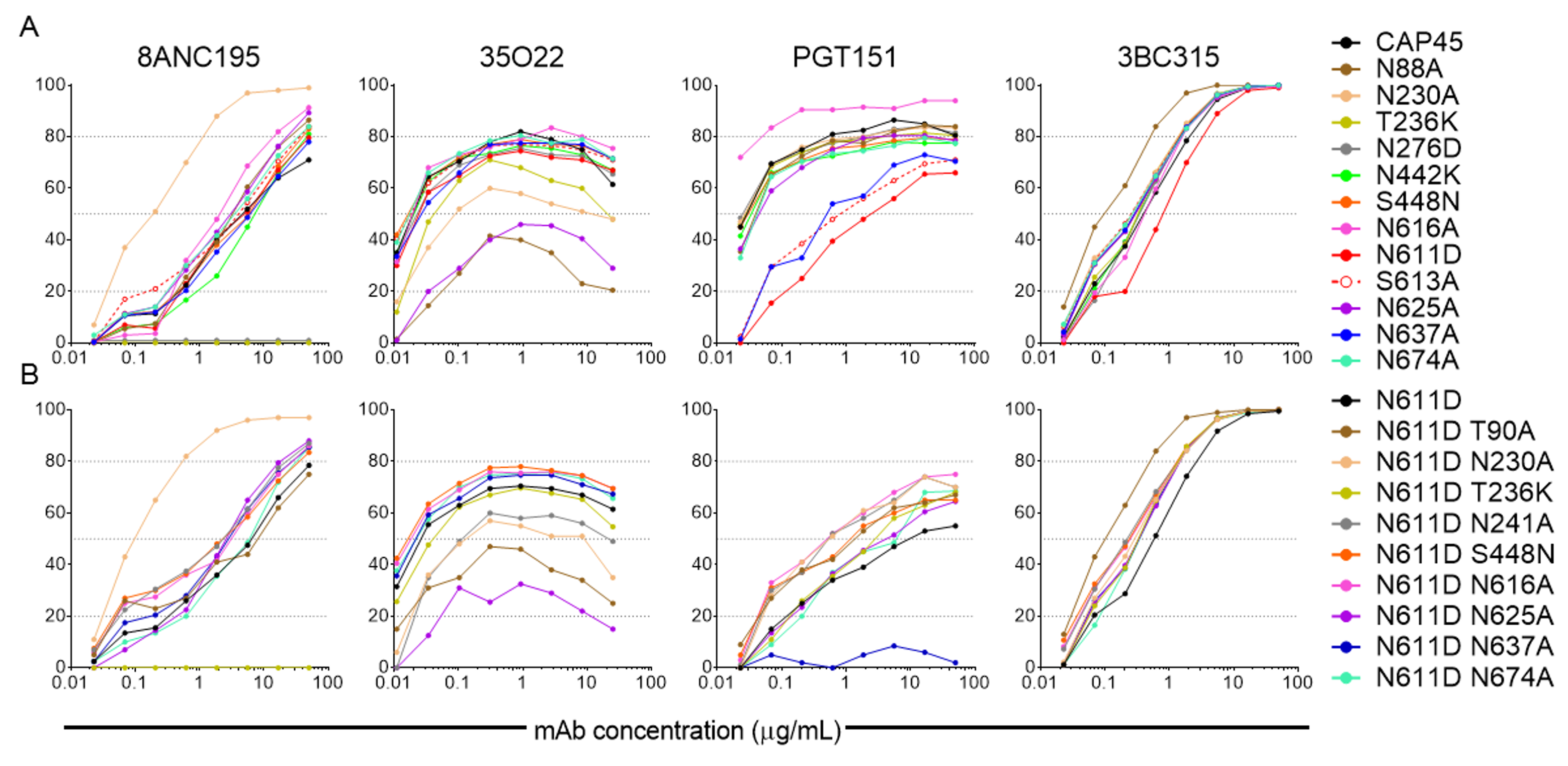

Supplement: S6 Fig — (A) Neutralization of CAP45 by broadly neutralizing antibodies that target the gp120-gp41 interface, when compared to CAP248-2B epitope proximal glycan mutants. The S613A mutant that removed the glycan at N611 but maintains the amino acid side chain properties is shown with red dashed lines and open circles. Percentage inhibition is plotted on the y-axis versus CAP248-2B antibody concentration on the x-axis. (B) Neutralization of the CAP45 N611D single mutant, and various N611D including double glycan mutants, plotted as in A. (TIF) [file ppat.1006074.s006.tif]

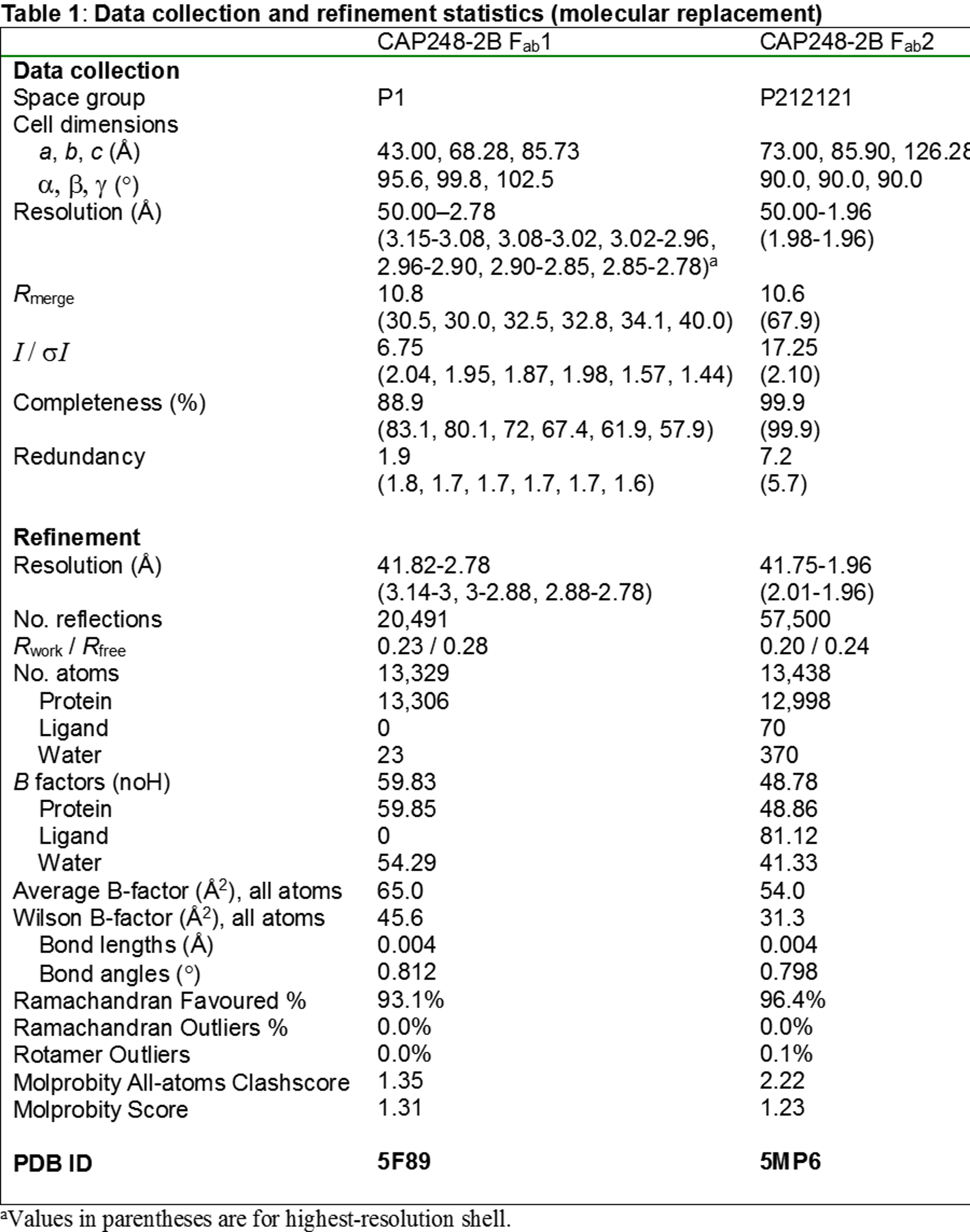

Supplement: S1 Table — Data collection and refinement statistics for the two unliganded CAP248-2B Fab crystal structures. (TIF) [file ppat.1006074.s007.tif]
